# Role of ribosome recycling factor in natural termination and translational coupling as a ribosome releasing factor

**DOI:** 10.1371/journal.pone.0282091

**Published:** 2023-02-24

**Authors:** Yoshio Inokuchi, Fabio Quaglia, Akikazu Hirashima, Yoshihiro Yamamoto, Hideko Kaji, Akira Kaji

**Affiliations:** 1 Faculty of Science and Engineering, Department of Biosciences, Teikyo University, Utsunomiya, Japan; 2 Department of Microbiology, Perelman School of Medicine, University of Pennsylvania, Philadelphia, Pennsylvania, United States of America; 3 Hyogo College of Medicine, Nishinomiya, Japan; 4 Department of Biochemistry and Molecular Biology, Jefferson Medical College, Philadelphia, Pennsylvania, United States of America; Bhabha Atomic Research Centre, INDIA

## Abstract

The role of ribosome recycling factor (RRF) of *E*. *coli* was studied *in vivo* and *in vitro*. We used the translational coupling without the Shine-Dalgarno sequence of downstream ORF (d-ORF) as a model system of the RRF action in natural termination of protein synthesis. For the *in vivo* studies we used the translational coupling by the adjacent coat and lysis genes of RNA phage GA sharing the termination and initiation (UAAUG) and temperature sensitive RRF. The d-ORF translation was measured by the expression of the reporter *lacZ* gene connected to the 5’-terminal part of the lysis gene. The results showed that more ribosomes which finished upstream ORF (u-ORF) reading were used for downstream reading when RRF was inactivated. The *in vitro* translational coupling studies with 027mRNA having the junction sequence UAAUG with wild-type RRF were carried out with measuring amino acids incorporation. The results showed that ribosomes released by RRF read downstream from AUG of UAAUG. In the absence of RRF, ribosomes read downstream in frame with UAA. These *in vivo* and *in vitro* studies indicate that RRF releases ribosomes from mRNA at the termination codon of u-ORF. Furthermore, the non-dissociable ribosomes read downstream from AUG of UAAUG with RRF *in vitro*. This suggests that complete ribosomal splitting is not required for ribosome release by RRF in translational coupling. The data are consistent with the interpretation that RRF functions mostly as a ribosome releasing factor rather than ribosome splitting factor. Additionally, the *in vivo* studies showed that short (less than 5 codons) u-ORF inhibited d-ORF reading by ribosomes finishing u-ORF reading, suggesting that the termination process in short ORF is not similar to that in normal ORF. This means that all the preexisting studies on RRF with short mRNA may not represent what goes on in natural termination step.

## Introduction

Ribosome recycling factor was first found to be an enzyme that releases ribosomes from mRNA for a new cycle of translation. Hence, the factor was originally called “ribosome releasing factor (RRF)” [1] and the gene, *frr*, and the promoter position were identified near 4 minutes in the *E*. *coli* genetic map [2]. For early review see [3,4]. Later, the factor was re-named “ribosome recycling factor” [5] because its function is to recycle the used ribosomes by releasing them from mRNA and tRNA [6]. One additional function, splitting of ribosomes of the PoTC (post-termination complex) during recycling, was discovered through kinetic analysis of formation of oligopeptides [7]. The ribosome splitting activity of RRF was confirmed later by three independent laboratories [8–10]. The recycling process is an essential step in all organisms so far studied [5,11–14].

When RRF is absent, ribosomes stay at the termination codon and start translating from the next codon [15]. This is called “unscheduled translation” [16] because translation beyond the termination codon is not scheduled. This is somewhat similar to the term re-initiation but we prefer to use the term “unscheduled translation” specifically referring to translation due to failure of ribosome recycling. There is another factor, IF3, that helps dissociation of 70S ribosomes [17,18]. It is likely that in the absence of RRF, the role of IF3 [19] as the second splitting factor would become important. RRF is also suggested to function as an agent dealing with stalled ribosome [20].

RRF has a striking structural similarity to tRNA [21]. Studies suggest that RRF binds to 70S ribosomes covering the A/P site [22–26] and moves within the inter-ribosomal space to the P-site [27–29] like tRNA. The RRF reaction is dependent on GTP [30–33] just like the movement of tRNA during translocation. However, the orientation of ribosome-bound RRF is upside-down compared to that of tRNA. In a recent publication using time-resolved cryo-EM, which makes it possible to visualize short-lived intermediates, the mRNA was never released from the ribosome due to proximity of the Shine-Dalgarno (SD) sequence to the termination codon [29]. Another paper utilized a novel single-molecule fluorescence technique to visualize the interactions of molecules in ribosome recycling [34]. However, their results showed a very late and slow release of 30S ribosomal subunit from the mRNA, which may be due to the presence of a SD sequence near a very short ORF. A recent report grants an interesting view of the crystal structure of a ribosome with two tRNAs, RRF and EF-G [35]. The role of this complex in the RRF reaction remains to be elucidated.

The overlapping sequence at the border region between two adjacent ORFs in a polycistronic mRNA was first discovered at the border region of *trpB* and *trpA* [36]. It was thought to be peculiar to the case of translational coupling where a certain ratio of a pair of proteins is required for the function of two proteins. However, the complete sequencing of bacterial genomes suggests that such sequences are common in the inter-cistronic region in an operon in *E*. *coli* [37]. We define translationally coupled ORFs as a pair of ORFs where reading of the downstream ORF (d-ORF) is performed, totally or partially, by the ribosome(s) which have completed translation of the upstream ORF (u-ORF) [38]. In our preceding publication, we showed that, in RNA phage GA, the translation of the lysis protein gene (d-ORF) without an SD sequence was performed strictly by the ribosomes that translated the coat protein gene (u-ORF) [39]. The deletion and base substitution analyses revealed that there were no sequences that allowed the expression of the downstream lysis gene independently of the translation of the upstream coat gene. During this process, the ribosomes accomplish the frameshift required by the overlapping initiation and termination codons.

In this paper, we investigated functions of RRF during the natural termination process using the translationally coupled system without the SD sequence of d-ORF. Our data, both *in vivo* and *in vitro*, are consistent with the interpretation that RRF releases ribosomes from the termination codon of the junction sequence, which allows the released ribosome to bind to the nearby initiation triplet of d-ORF. In the *in vivo* experiments, this basic behavior of ribosomes is influenced by the sequence surrounding the termination triplet. When u-ORF was very short, d-ORF reading was not observed at all. Furthermore, in the *in vitro* experiments, use of genetically conjugated ribosomal subunits [40] showed that the recycling of ribosomes at the junction sequence can occur without complete splitting of the 70S ribosomes of PoTC. This indicates that our original concept of RRF as the “ribosome releasing factor” [1,41] is correct in that the major function of RRF resides in the release of ribosomes from mRNA of PoTC.

## Results

### Translationally coupled ORF without the SD sequence for d-ORF gives a way to study the role of RRF at the termination step

The *in vivo* behavior of ribosomes that just finished termination step is not well understood. However, it is known that, in translational coupling, ribosomes which are finishing the translation of u-ORF (u-ribosomes) are responsible for the translation of d-ORF without SD [39,42]. The lysis gene of RNA phage GA is exclusively read by the ribosomes that have completed the reading of the upstream coat gene. This is achieved by the unique junction sequence UAAUG present between these two ORFs. On this basis, we describe here series of experiments indicating the role of RRF on the behavior of u-ribosomes by using such translational coupling.

**Fig 1:**
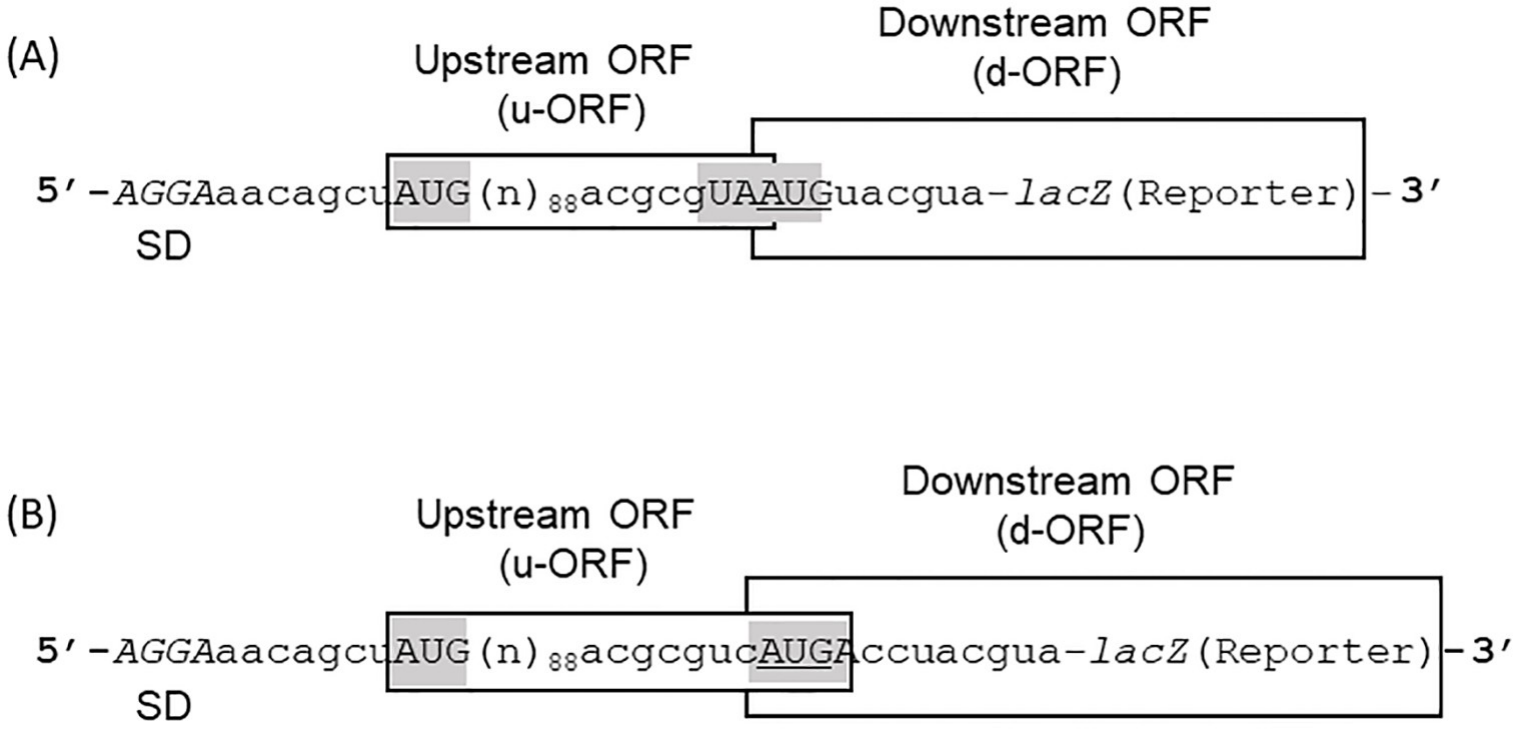
Representative diagrams of plasmids used in our experiments. Diagrams of plasmids (A) and (B) are shown. Initiation codon and termination codons are shown in capital letter and highlighted for clarity. AUG is the initiation codon for upstream open reading frame (u-ORF) and AUG is that for downstream open reading frame (d-ORF). The initiation codon for d-ORF is overlapped with the termination codon for u-ORF ((A) UAA and (B) UGA). u-ORF and d-ORF are boxed. Nucleotide sequence 5’-AGGA-3’ shown in italic represents a Shine-Dalgarno (SD) sequence of u-ORF. *LacZ* represents the reporter *lacZ*(Δ27) gene for β-galactosidase. n in the bracket represents a nucleotide and numbers next to the bracket represent the number of nucleotides in the bracket. The nucleotide sequence labelled (n)_88_ is referred to [39,43] for details.

To investigate the role of RRF in ribosome recycling *in vivo*, we constructed plasmids having the cDNA of GA phage including the region between the coat and lysis genes [39]. With these plasmids, the lysis gene was replaced by the reporter *lacZ* gene to study d-ORF expression precisely. Furthermore, to see whether the coupling system works only with the junction sequence UAAUG, we examined the sequence AUGA as well. As shown in Fig 1, a reporter *lac*Z gene (d-ORF) coding for β-galactosidase was connected to the C-terminal sequence of GA phage coat gene (u-ORF) through the junction sequence UAAUG in (A) or AUGA in (B). UAA and UGA are termination triplets of u-ORF. AUG is in frame with the *lacZ* gene. We define the reading frame of AUG as zero frame in this paper. The plasmids express the reporter gene through translational coupling. To control the RRF activity *in vivo*, we used the *E*. *coli* strain LJ14, which contained a temperature sensitive mutation of RRF (tsRRF). With this strain, tsRRF is active at 31°C while it is inactive at 37°C [16].

**Fig 2:**
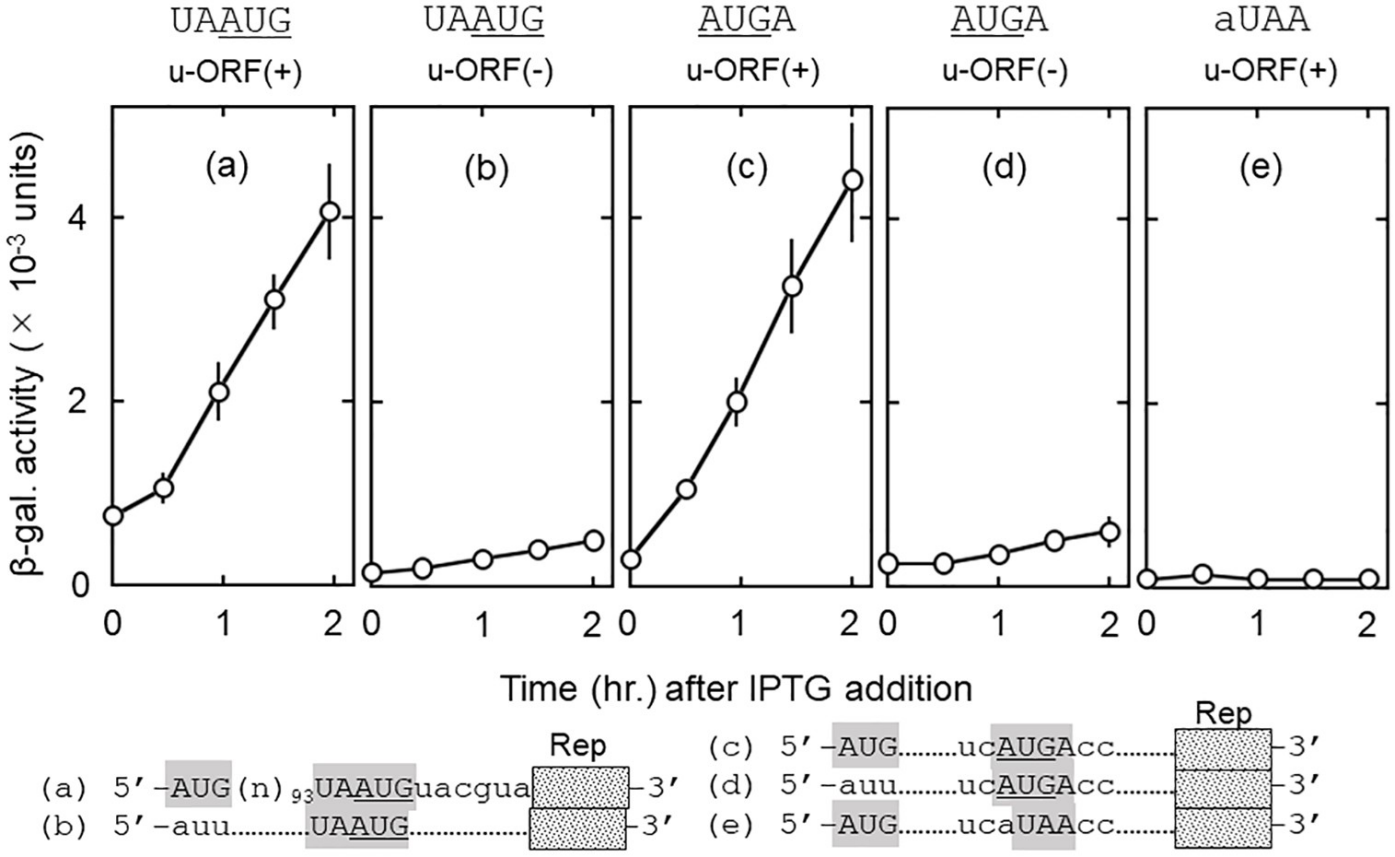
Downstream reading of translationally coupled ORFs is dependent on the presence of u-ORF and the initiation triplet of d-ORF. *E*. *coli* LJ14 cells harboring plasmids were grown at 28°C to a cell density of ~0.13 as described in Experimental procedures and IPTG (final 2 mM) was added to the culture for induction of the reporter *lacZ*(Δ27) gene. The culture was incubated for another two hours. At the indicated times after the addition of IPTG, an aliquot was taken and the β-galactosidase activity was measured. The junction sequences and presence (+) or absence (−) of u-ORF are shown above each panel. The nucleotide sequences around the junction sequence are shown at the bottom. Rep represents the reporter *lacZ*(Δ27) gene. Dotted lines (……) in (b) through (e) represent the same nucleotide sequences with that of (a) in the corresponding regions. Panels (a) and (c) represent the β-galactosidase synthesis through translational coupling. (b) and (d) are identical to (a) and (c), respectively, with the exception that AUG of u-ORF was changed to auu. (e) is identical with (c) with the exception that (e) has aUAA instead of AUGA. The reporter *lacZ* gene is in frame with AUG of UAAUG, AUGA and aUA of aUAA.

Fig 2 shows that our translational coupling system described above confirmed the following characteristics of the system by the coat-lysis genes pair. First, all ribosomes that translate d-ORF are derived from u-ribosomes. When u-ORF was lost (panels (b) and (d)), no d-ORF reading was observed with the junction sequences UAAUG (compare (b) with (a)) and AUGA (compare (d) with (c)). Free ribosomes are excluded from engaging in translation of d-ORF without SD. Second, u-ribosomes are released at the termination codon of u-ORF and re-bind to the initiation codon of d-ORF (frameshift). This is because the reading frame of u-ORF differs from that of d-ORF. Panel (e) indicates that triplet aUA (coding for isoleucine but not for methionine) did not function as the initiation codon for d-ORF (compare panel (e) with (c)). Third, u-ribosomes move both directions at the termination codon. As shown in panels (a) and (c), the released u-ribosomes bound to AUG situated downstream of the termination codon of u-ORF (panel (a)) as well as AUG situated upstream of that (c).

### *In vivo* evidence that RRF releases all ribosomes from the termination triplet

**Fig 3:**
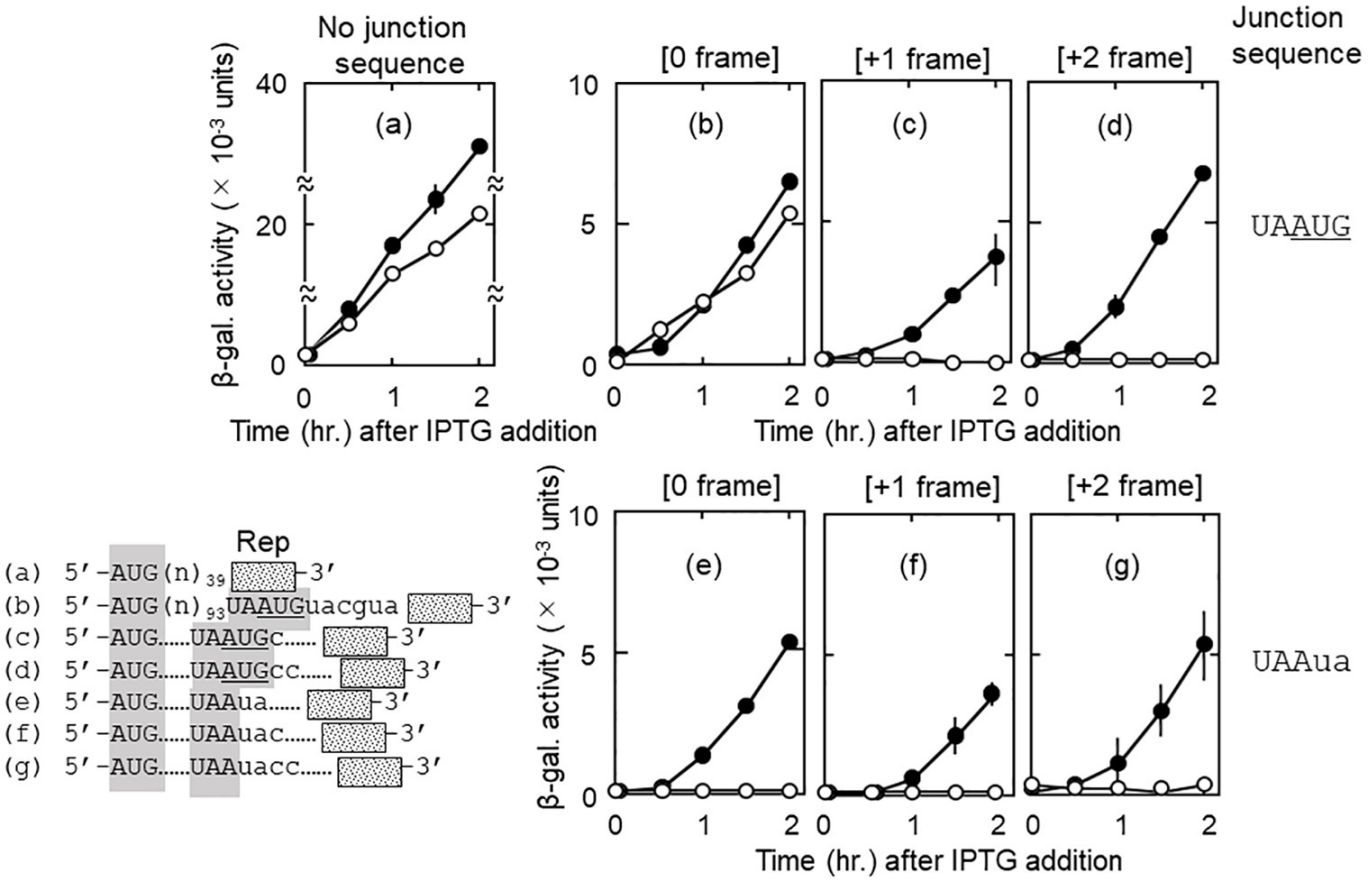
With heat inactivation of RRF, ribosomes remain on the mRNA and translate the downstream region in all three frames. Numbers in square brackets shown above each panel represent the reading frame of d-ORF counting from AUG or Aua of UAAUG or UAAua, respectively, shown on the right side of panels. Panel (a) (a control plasmid) has no junction sequence and represents the u-ORF reading. The scale of the β-galactosidase activity in panel (a) is four times larger than that of (b) through (g). Panel (b) has the junction sequence UAAUG and represents the β-galactosidase synthesis through the coupling mechanism. The reporter *lacZ*(Δ27) gene of (b) is in zero frame (in frame with AUG of UAAUG). Panels (c) and (d) represent the cases in which the reporter gene is in +1 (c) and +2 (d) frame, respectively, counting from AUG of UAAUG. Panels (e), (f) and (g) have the sequence UAAua instead of UAAUG. Dotted lines (……) represent the same nucleotide sequences with that of (b) in the corresponding regions.

We have demonstrated that RRF releases mRNA from PoTC during ribosome recycling *in vitro* [1,24,41]. This predicts that, in our *in vivo* translational coupling system, all u-ribosomes would be released by RRF from UAA of the junction sequence UAAUG. Some of the released u-ribosomes would re-bind to AUG of UAAUG, followed by d-ORF (reporter gene) reading. To examine this prediction, we constructed three kinds of plasmids in which the reporter *lacZ* gene was connected to the junction sequence in three reading frames to observe all frames of translation. We then compared the amounts of ribosomes engaging in downstream reading (β-galactosidase activity) in the presence and absence of RRF. Our prediction that RRF releases all ribosomes from PoTC *in vivo* was fulfilled as shown in Fig 3 and Table 1.

**Table 1:** Heat inactivation of RRF makes more u-ribosomes read downstream.

Junction sequence	Culture temp.	RRF activity	% of u-ribosomes which translate reporter gene at 2 hours after IPTG addition*			
			0 frame (a)	+1 frame (b)	+2 frame (c)	All frames (a) + (b) + (c)
**UA** **AUG**	**28°C**	**+**	24.7	0.2	0.6	25.5
**UA** **AUG**	**39°C**	**−**	20.7	12.1	21.5	54.3
**UAAua**	**28°C**	**+**	0.4	0.3	1.5	2.2
**UAAua**	**39°C**	**−**	17.2	11.7	17.2	46.1

*: Values were expressed as the percentage of u-ORF reading. For example, the value (24.7%) shown in line 1, column 4 was calculated as follows: 24.7% = 100 × {5,200 units (Fig 3 panel (b), open circle at 2 hr.) ÷ 21,000 units (Fig 3 panel (a), open circle at 2 hr.)}.

In this figure, with the junction sequence UAAUG, d-ORF reading (β-galactosidase activity) was observed in 0 frame (panel (b)) but not in +1 (c) and +2 (d) frames in the presence of RRF (open circles). This is because u-ribosomes were released at UAA and bound back specifically to AUG of UAAUG. On the contrary, in the absence of RRF activity (closed circles), d-ORF reading was also observed in +1 (c) and +2 (d) frames. This indicates that u-ribosomes remained on the mRNA and re-initiated translation in all three frames. Furthermore, when AUG of UAAUG was changed to Aua (Fig 3 panels (e), (f) and (g)), d-ORF reading was not observed in any frame in the presence of RRF (open circles), indicating that u-ribosomes were released from RNA. However, in the absence of RRF activity (closed circles), d-ORF reading was observed in every reading frame, indicating that u-ribosomes remained on the mRNA.

To estimate how much of u-ribosomes would stay on the mRNA, percentages of the u-ribosomes engaged in d-ORF reading were calculated from Fig 3 and summarized in Table 1. It is clear from this table (column 7, All frames) that more u-ribosomes read downstream in the absence of RRF activity than in the presence of that (UAAUG: 54.3% vs 25.5%, UAAua: 46.1% vs 2.2%). However, the total amount of d-ORF readings (54.3% and 46.1%) were less than 100% of u-ORF reading. This is because, at the non-permissive temperature for tsRRF, the ribosomes that completed ORF reading often slide toward the 3’ direction without translation and resume translation at random [16]. Protein products made by ribosomes that resume translation within the reporter *lacZ* gene would not have the enzyme activity and will not be counted in our assay. It is therefore assumed that, in the absence of RRF, the actual number of mRNA-bound u-ribosomes engaged in downstream reading would be higher than the value shown in Table 1. From these considerations, we conclude that most, if not all, ribosomes remained on mRNA in the absence of RRF activity.

**Fig 4:**
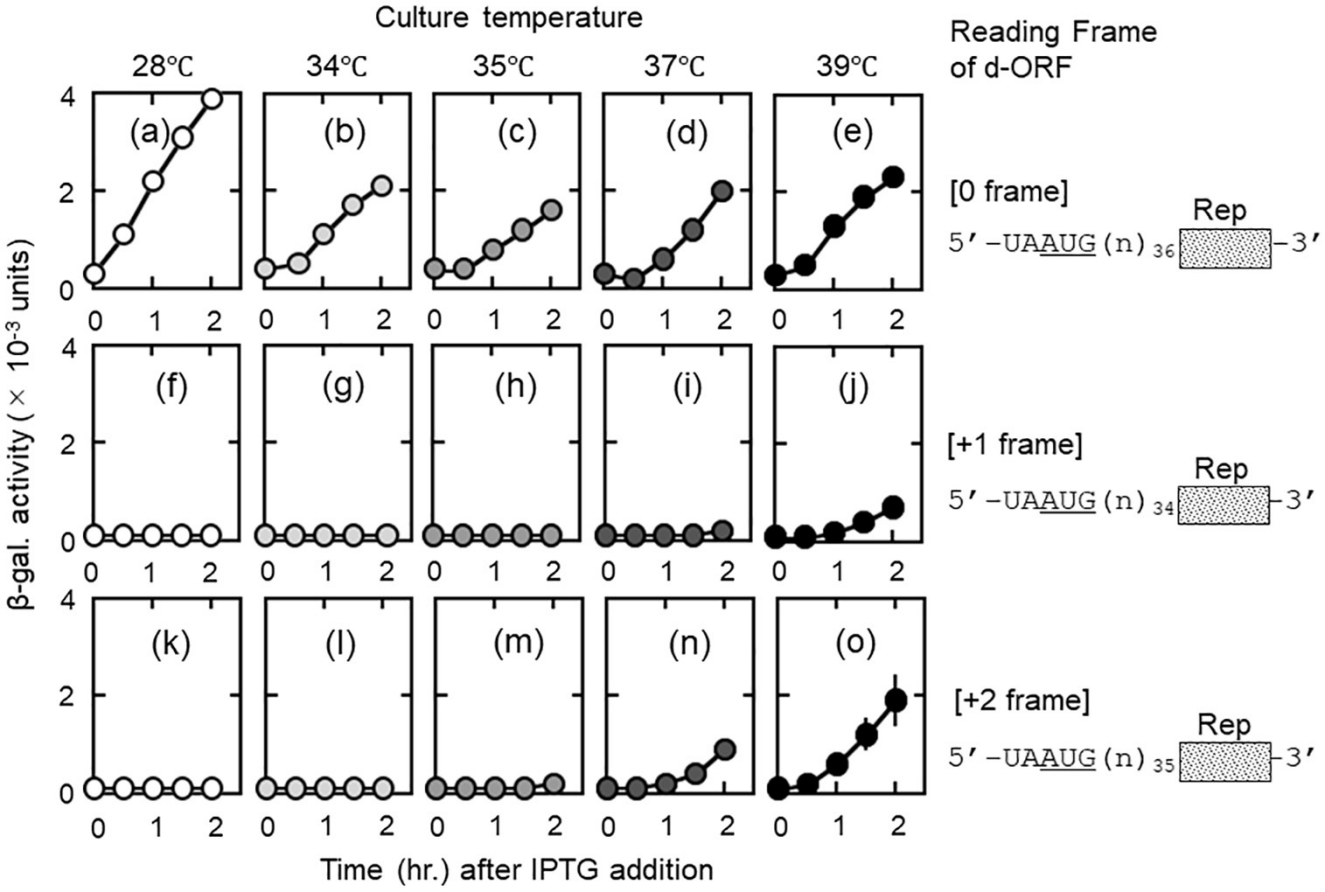
Both high culture temperature and loss of RRF function are required for thermal frameshifts to all frames. LJ14 cells grown at 28°C were divided into 5 parts and further incubated at various temperatures. Numbers in square brackets shown on the right side of panels represent the reading frame of d-ORF counting from AUG of the junction sequence UAAUG. In panels (a)—(e), AUG of UAAUG is in frame with the reporter *lacZ*(Δ27) gene. In (f)—(j) and (k)—(o), AUG of UAAUG is out of frame with the reporter gene. The nucleotide sequences labelled (n)_n_ are 5’-gguctcaaagcaaaacacaaggaaaacctcgcgaaauacgua-3’ (panels (a)–(e)), 5’-gguctcaaagcaaaacacaaggaaaacctcgcgauacgua-3’ ((f)–(j)), and 5’-gguctcaaagcaaaacacaaggaaaacctcgcgaauacgua-3’ ((k)–(o)). At 39°C, the temperature is high enough to inactivate RRF and cause thermal frameshift, which is shown by reading at all three frames (panels (e), (j) and (o)).

The effects of temperature on wild-type RRF regarding the reporter gene expression have been reported [16]. In that paper, we have shown that, with normal ORF, downstream reading was not observed when the culture temperature was shifted from 31 to 39°C. Furthermore, LJ14 cells harboring plasmid pPEN907 carrying wild-type *frr* showed wild-type phenotype at the permissive and non-permissive culture temperature (Fig 4 panel B of [16]). These results indicate that, with wild-type RRF, the change of culture temperature does not cause the change of the amount of downstream reading in our system.

### At the non-permissive temperature of tsRRF, ribosomes remaining at the termination codon undergo frameshift to all frames

Structural studies [24,28,29,44] showed that the natural substrate of RRF does not have a peptidyl group which functions to stabilize the elongating ribosomes. Based on this consideration, we postulated that the ribosome, by the inactivation of tsRRF, would stay at the UAA codon without stabilization by peptidyl group. Because of the high culture temperature (39°C) used to inactivate tsRRF, these ribosomes would then start vibrating (thermal vibration) at UAA, and would be frameshifted to all frames. This prediction was fulfilled as shown in Fig 3 (closed circles).

In this figure, we show that, at 39°C where RRF activity was absent, all frames were read (panel (b) to (g)). This indicates that ribosomes which finished u-ORF reading at the UAA frameshifted to all frames. We should recall that, without RRF, ribosomes read downstream only in frame with the termination codon *in vitro* [15]. If ribosomes behave similarly *in vivo* as *in vitro*, when we inactivate tsRRF, we would see reading of the downstream reporter gene only in frame with UAA. In other words, ribosomes would read +1 frame only (panels (c) and (f)), closed circles). On the contrary, we saw the downstream readings in all frames. This is because, due to the high culture temperature, temperature-dependent frameshift to all frames took place for ribosomes that remained on the UAA.

### Both inactivation of RRF and high culture temperature are necessary for frameshift to all frames

We postulate that there are two elements influencing frameshift; high culture-temperature and loss of RRF activity. To sort out the temperature effect and the effect of the loss of RRF activity on frameshift at the junction sequence UAAUG, we tested the system at various temperatures for frameshift to all frames or to zero frame only (from UAA to AUG). The results are shown in Fig 4.

As shown in panels (c), (h), and (m), only frameshift to zero frame (AUG) occurred at 35°C by ribosomes released by remaining RRF activity. At 37°C, where tsRRF is inactivated [16], preferential reading of AUG began to wane (panel (n)) because temperature-dependent vibration of ribosomes occurred. However, panels (d) and (i) show that even at 37°C the remaining RRF activity is sufficient to release ribosomes from UAA to bind back to AUG. Since most of the ribosomes read the frame starting from AUG, temperature-dependent frameshift to all frames was not observed. At 39°C, frameshift to all three frames was observed, indicating that RRF activity was lost resulting in loss of AUG-specific reading. Frameshift to all frames due to high culture temperature begins here. Therefore, we conclude that both complete inactivation of RRF and a high culture temperature are required for temperature-dependent frameshifting. In fact, *in vitro* experiments described in the later section of this paper show that removal of RRF alone does not induce frameshift to all frames. The above conclusion is also consistent with the fact that no published paper has shown any frameshift at high temperature using wild-type *E*. *coli*.

### The distance between AUG and UAA in the junction sequence influences d-ORF reading only in the presence of RRF: Further support for RRF-dependent release of ribosomes from PoTC

**Fig 5:**
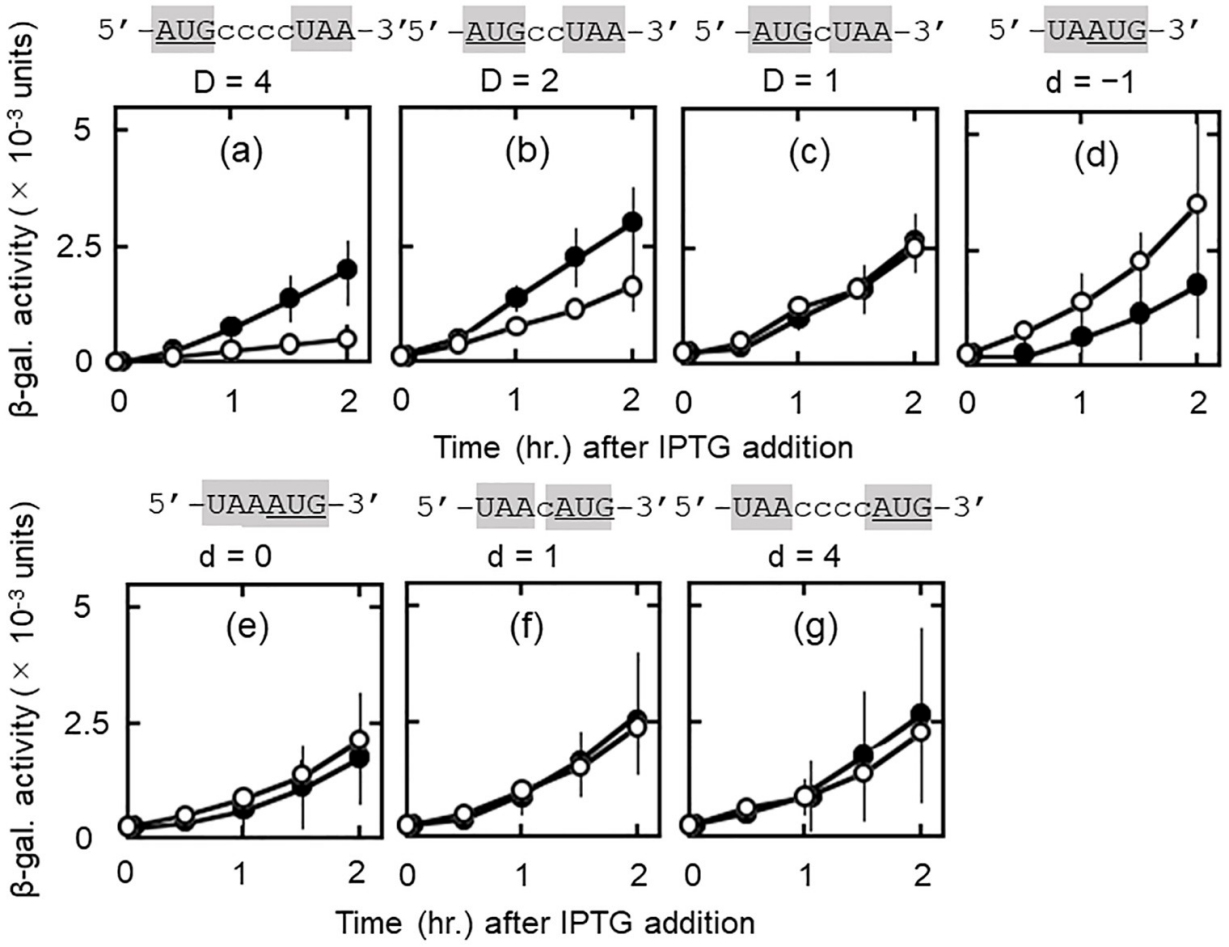
The distance between AUG and UAA of the junction sequence influences downstream reading only in the presence of RRF. Nucleotide sequence and the distance (D and d) between AUG and UAA at the junction regions are indicated above each panel. AUG is the initiation codon of d-ORF and in frame with the reporter *lacZ*(Δ27) gene. UAA is the termination codon of u-ORF. D represents distance in nucleotides when AUG is upstream of UAA while d represents distance when AUG is downstream of UAA. The minus sign (−) in panel (d) indicates that UAA and AUG are overlapped by one nucleotide (d = −1). Cases where AUG is located upstream of UAA with distance 3 and 0 nucleotides (D = 3 and 0) are not shown because AUG is in frame with downstream UAA.

Up to this point, we presented the cases where termination codon of u-ORF and initiation codon of d-ORF overlap (for example, UAAUG). In the experiments shown in Fig 5, we studied the effect of the distance between UAA and AUG on d-ORF reading. We reasoned that ribosomes can start reading from any position on mRNA as long as it remains attached to the mRNA. On the other hand, ribosomes once released from the mRNA have to seek AUG to re-start the translation. Therefore, one would expect that the distance would make difference on the downstream reading only in the presence of RRF. The amount of d-ORF reading would decrease as the distance between UAA and AUG increases. This prediction was fulfilled as described below.

As shown in panels (a) through (c) of Fig 5, when AUG was situated upstream of UAA, there was a distinct effect of the distance (shown as D) between AUG and UAA on d-ORF reading (open circles). The β-galactosidase activity in (a) (D was 4 nucleotides) was about 20% of that in (c) (D was one nucleotide) at 2 hrs. after IPTG addition. The amount of d-ORF reading decreased as the distance between AUG and UAA increased. These are cases where ribosomes must move backwards from 3’ to 5’ direction (against the natural direction of ribosome movement) to bind to AUG.

In contrast, in panels (d) through (g) (open circles), when the released ribosomes must bind to AUG situated downstream from UAA, the expression of the reporter gene was approximately equal in all cases regardless of the distance (d equals -1, 0, 1 and 4 nucleotides). We interpret that the moment of inertia of the ribosomes moving toward the 3’ direction masks the effect of distance when the distance is only a few nucleotides.

What is the effect of the distance and direction of ribosome movement in the absence of RRF? In these cases, the ribosomes which completed u-ORF reading were allowed to move toward the 3’ direction on the mRNA and resume translation from random places [16]. Therefore, for the ribosomes remaining at UAA, the distance between AUG and UAA and the relative position of these triplets would have no effect on d-ORF reading. This was true as shown in panels (a) through (g) (closed circles). We conclude from Fig 5 that ribosomes were released at UAA from mRNA of PoTC by RRF and rebound to AUG of the junction sequence.

### The SD sequence near the junction region attracts mRNA-bound ribosomes as well as released ribosomes after termination

**Fig 6:**
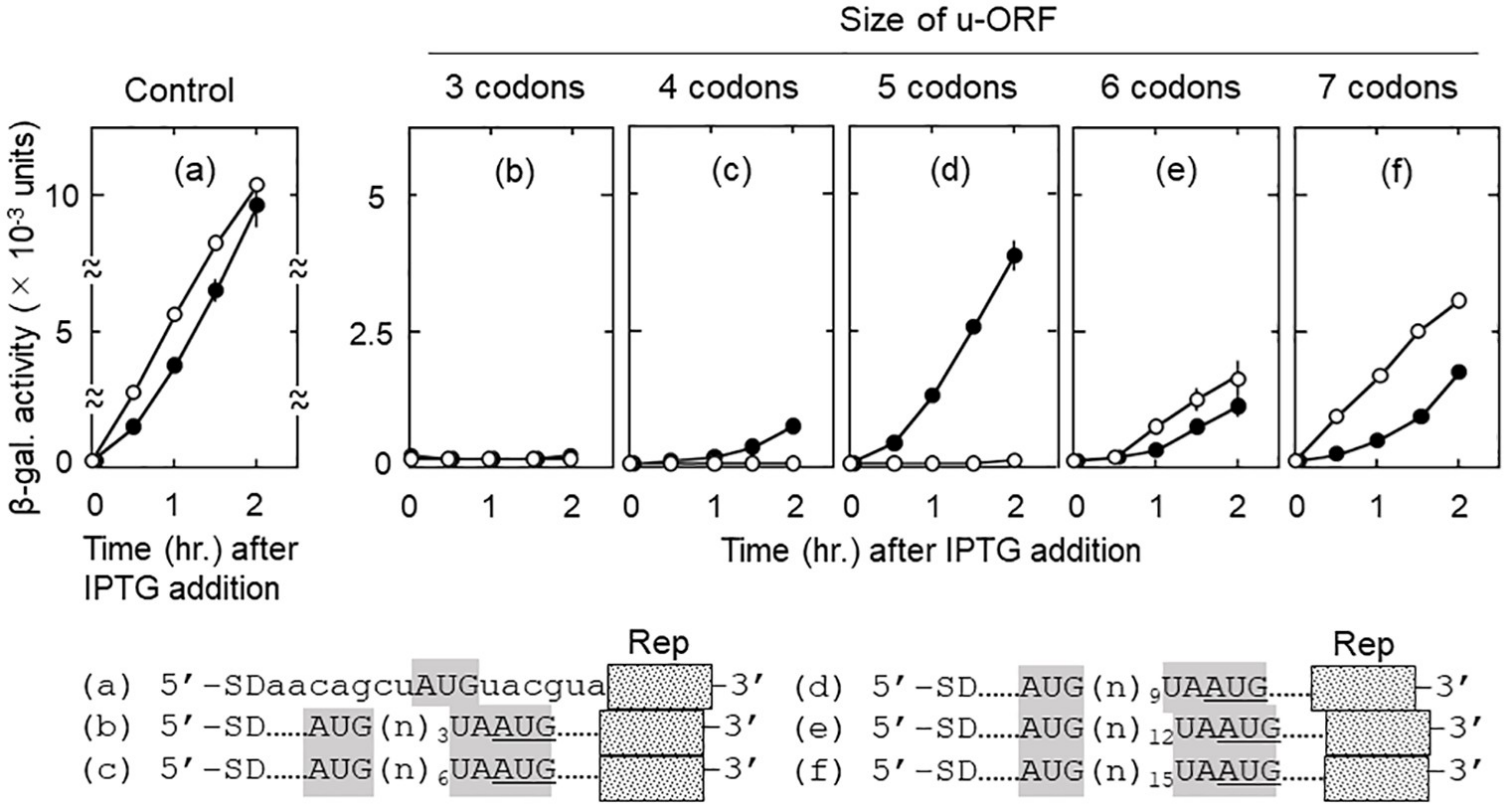
The smaller the size of u-ORF, the less the d-ORF reading. Numbers of codons in u-ORF are shown above each panel. SD represents the SD sequence (5’-AGGA-3’). AUG of the junction sequence UAAUG is in frame with the reporter *lacZ*(Δ27) gene. n in the bracket represents a nucleotide and numbers next to the bracket show the number of nucleotides in the bracket. The nucleotide sequences around the junction sequence are shown at the bottom. Dotted lines (……) in (b) through (e) represent the same nucleotide sequences with that of (a) in the corresponding regions. Panel (a) has no junction sequence and represents the u-ORF reading. The nucleotide sequences labelled (n)_n_ are 5’-aca-3’, 5’-accauc-3’, 5’-accaugauu-3’, 5’-accaugauuacc-3’, and 5’-accaugauuacgaau-3’ for panel (b), (c), (d), (e) and (f), respectively. Dotted lines (……) represent the same nucleotide sequences with that of panel (a) in the corresponding regions.

In most of studies carried out *in vitro* to investigate the RRF function, mRNA contains the SD sequence near the termination triplet [8,29,31,34]. This is because the termination complex used was prepared with short mRNA. This results in the termination complex near SD. Since there is no SD near the *natural* termination triplet, we decided to elucidate the effect of SD on the behavior of ribosomes in the translational coupling between a short u-ORF with SD and d-ORF without SD. The ribosomes released from u-ORF would be attracted by the SD to re-read mostly the short u-ORF. Therefore, one would expect no reading of the d-ORF. In other words, the size of u-ORF is important for determining the amount of d-ORF reading by the ribosomes that have finished reading the u-ORF. A short u-ORF would abolish the expression of the reporter gene connected to the d-ORF. On the other hand, in the absence of RRF activity, u-ribosomes would be less influenced by SD because they are bound to mRNA. These expectations were fulfilled as shown in Fig 6.

**Fig 7:**
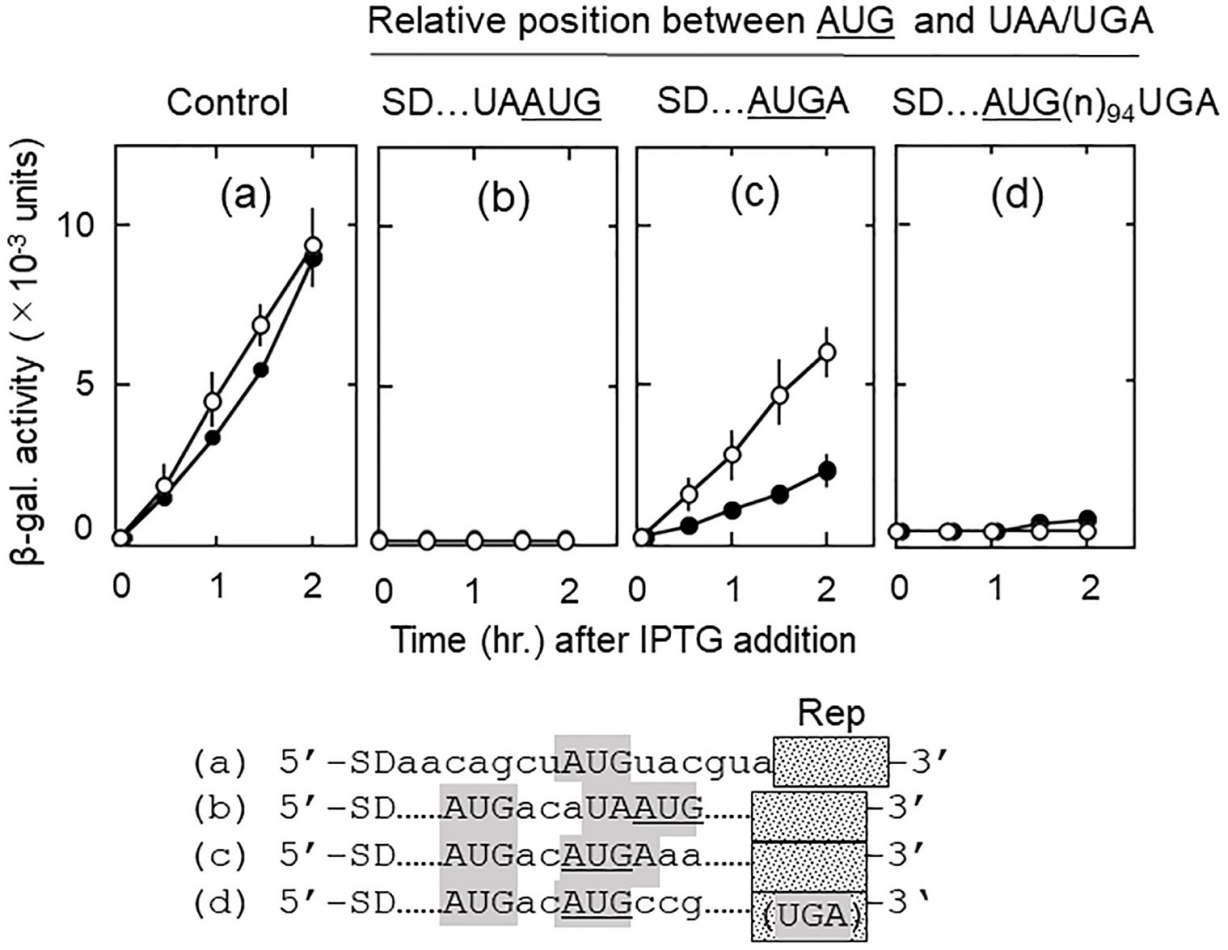
The relative positions of the initiation and termination codons in relation to the position of SD influences the d-ORF reading. Numbers of codons in u-ORF are shown above each panel. SD represents the SD sequence (5’-AGGA-3’). UAA and UGA are the termination codons of u-ORF. AUG is the initiation codon of d-ORF and in frame with the reporter *lacZ*(Δ27) gene. Dotted lines (……) represent the same nucleotide sequences with that of the plasmid of panel (a) in the corresponding regions. Panel (a) has no junction sequence representing the u-ORF reading. In panel (b), AUG is downstream of UAA. In (c), AUG is upstream of UGA. In (d), termination codon UGA of u-ORF is 94 nucleotides far from AUG and exists within the reporter gene.

In Figs 6 and 7, we designate the initiation signal of u-ORF as AUG and that of d-ORF as AUG. The termination codons of u-ORF are UAA or UGA, and we assume that UAA and UGA work similarly in all cases. As shown in Fig 6 panels (b), (c) and (d) (open circles), when u-ORF was shortened to three to five codons, there was no reporter gene expression. This suggests that all the ribosomes released by RRF from the termination triplet UAA of u-ORF were attracted by SD of the short u-ORF and re-read from the AUG to translate u-ORF but not d-ORF. On the other hand, when u-ORF was more than six codons, d-ORF was read ((e) and (f), open circles). This is because ribosomes released by RRF from UAA rebound to nearby AUG. These data suggest that, when u-ORF is more than six codons, the pulling force of the SD is weak enough for released ribosomes to be attracted by SD.

In the absence of RRF (closed circles), the size of u-ORF also has a similar effect on d-ORF reading. With 4, 5, 6 and 7 codons of u-ORF, the d-ORF was read (Fig 6 panels (c), (d), (e) and (f)). When u-ORF was shortened to three codons, d-ORF was not read (panel (b)). These data demonstrate that SD also influences the behavior of ribosomes remaining on the mRNA after termination. It should be mentioned that the expression of d-ORF was higher in 5 codon u-ORF (d) than in 6 codon u-ORF (e). This is contradicting with our expectations. We discuss this difference in Discussion section. The effect of SD was much stronger on ribosomes released by RRF than on ribosomes that remained on mRNA. This is because, in the presence of RRF (open circles), the effect was observable with 5 codon u-ORF (panel (d)). On the other hand, ribosomes remaining on the mRNA due to the loss of RRF showed the effect of SD with 3 codon u-ORF ((b), closed circles).

### Relative positions of the start and stop codons of the junction sequence with respect to SD determine the amount of d-ORF reading

In addition to the size of u-ORF, the relative positions of the start and stop codons of the junction sequence with respect to the SD sequence of short u-ORF should also determine the effect of SD on ribosomal behavior. We call this the directional effect of SD. As described in the preceding chapter, RRF-released ribosomes should be attracted toward upstream by the SD. Therefore, if the initiation codon of the d-ORF is upstream of the termination codon of the u-ORF, SD will help the released ribosomes move toward the initiation codon of the d-ORF and bind to it resulting in d-ORF reading. We predict that AUG of d-ORF situated upstream of UAA or UGA of u-ORF would stimulate d-ORF reading.

This expectation was met as shown in Fig 7. In panel (c) (junction sequence AUGA), where the initiation codon AUG was on the SD side of the termination codon UGA, there was a significant amount of d-ORF reading (open circles). This is because released ribosomes bind to the initiation triplet AUG on the way toward the SD. On the contrary (panel (b) (UAAUG)), there was no d-ORF reading when the initiation codon AUG was on the opposite side of SD of the termination codon UAA. The significant d-ORF reading (c) was not due to mRNA-free ribosomes attracted by SD, because when the termination codon UGA was moved 94 nucleotides downstream away from the initiation codon AUG, no d-ORF reading was observed (d).

In the absence of RRF (Fig 7, closed circles), we also observed the directional effect of SD on d-ORF reading by ribosomes that remained on the mRNA. As shown in panels (b) (junction sequence UAAUG) and (c) (AUGA), downstream reading was observed when AUG was on the side of SD (c) but not when AUG was on the opposite side of SD (b). These data support the idea that a nearby SD attracts RRF-released ribosomes as well as mRNA-bound ribosomes.

### *In vitro* evidence suggests that RRF acts as a ribosome releasing factor in translational coupling without SD of d-ORF

In this paper, we emphasize the importance of RRF action on the release of ribosomes from the PoTC. As a model, we chose translational coupling because the u-ribosomes must be used for reading from the initiation triplet of d-ORF without the SD sequence. This is accomplished by releasing u-ribosomes from the termination codon followed by rebinding of the ribosomes to the nearby initiation triplet of d-ORF. During this process, frameshift may occur. We investigated the essential nature of the RRF action in translational coupling as a frameshift causing agent in the *in vitro* system as described in this section.

**Fig 8:**
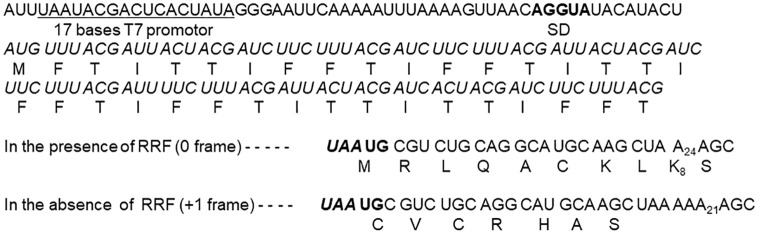
027mRNA sequence and translated peptides. Two ways of downstream reading that depend on the presence or absence of RRF are shown. u-ORF is in italic. SD and the junction sequence are in bold. Amino acid residues corresponding to the triplets of the reading frames are shown in single capital letters.

**Fig 9:**
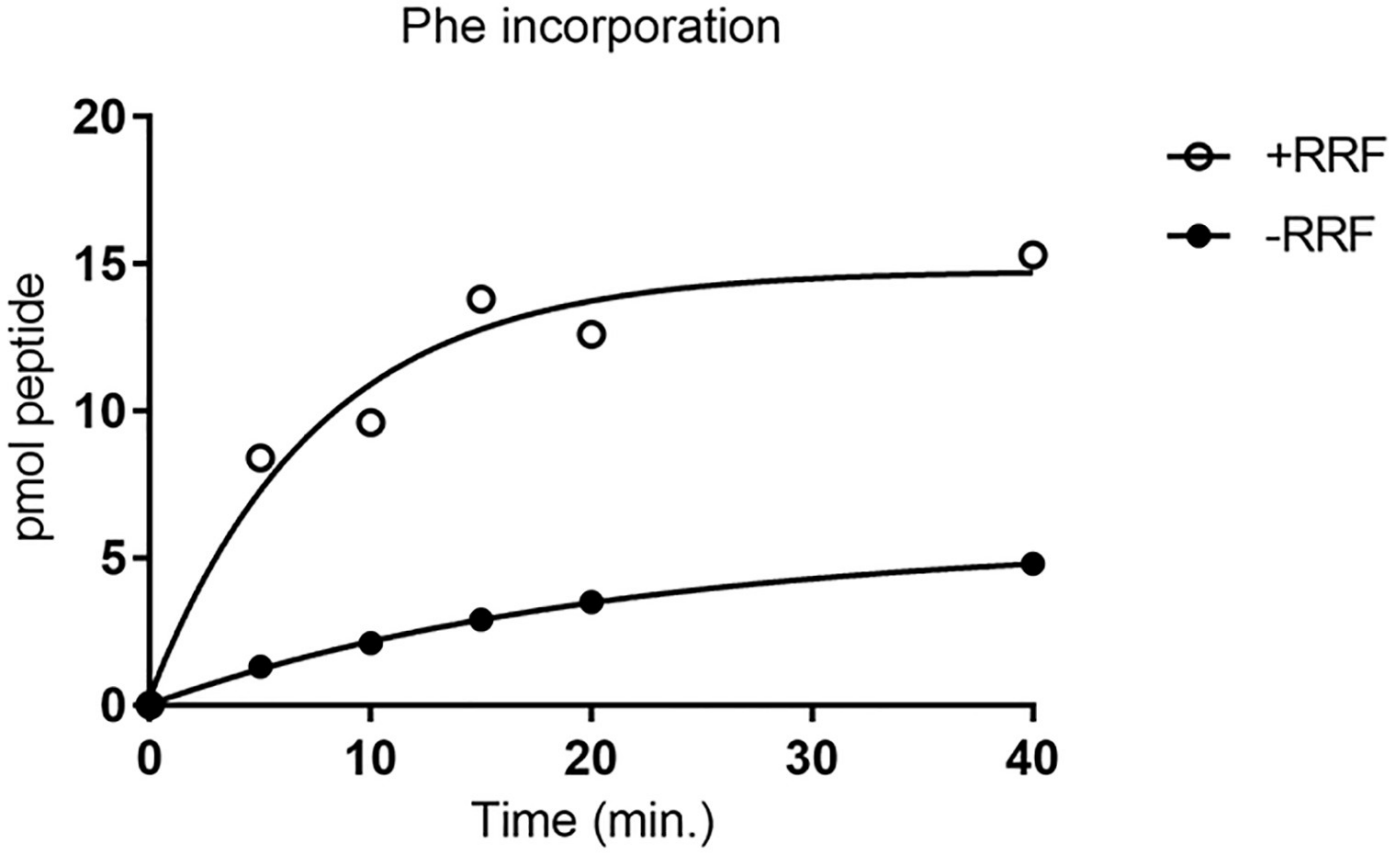
u-ORF translation is stimulated by RRF. u-ORF reading was measured by incorporation of [^3^H]-Phenylalanine coded for by UUU and UUC codons into hot TCA insoluble materials. Reaction mixtures were as described in Materials and Methods. The concentration of Mg^2+^ was 4 mM.

**Fig 10:**
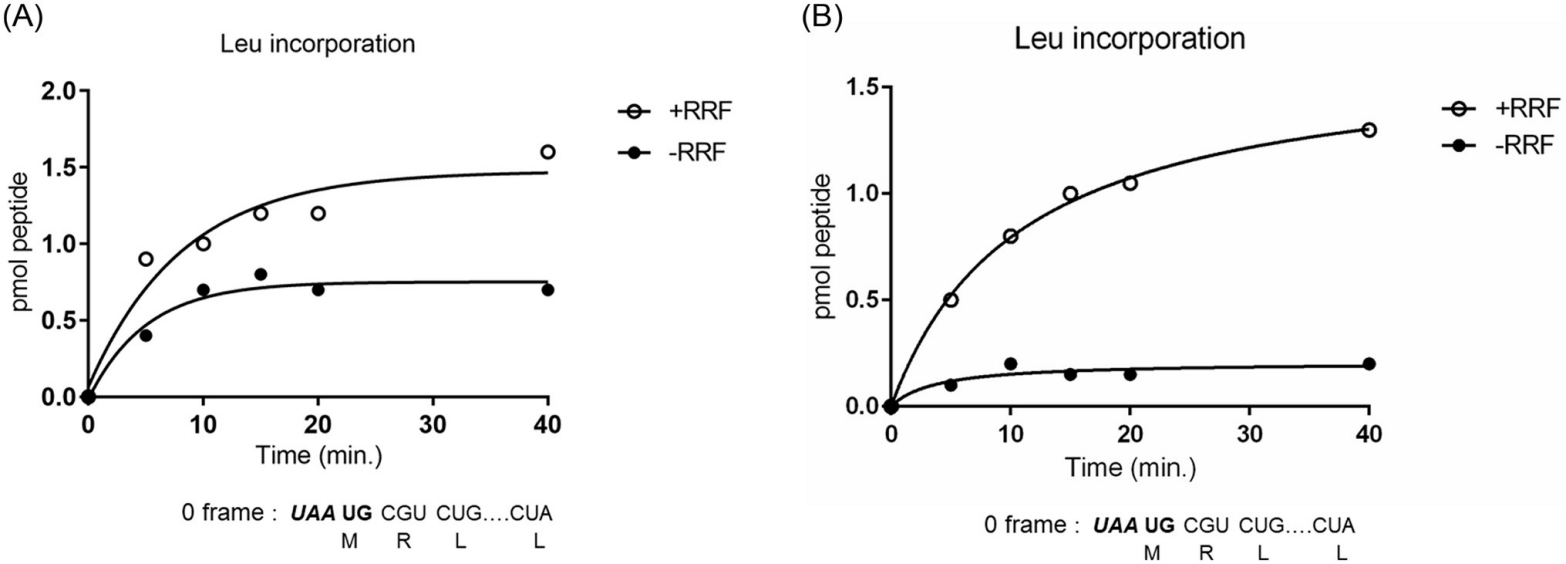
RRF stimulates translational coupling. **A** RRF stimulates downstream reading in frame with ***A*UG**. Downstream reading was measured by incorporation of [^14^C]-Leucine coded for by CUG and CUA codons in frame with AUG. Reaction mixture was identical to that of Fig 9 except for labeled amino acid (leucine). Mg^2+^ concentration was 4 mM. **B** At high Mg^2+^ concentration, downstream reading in frame with ***A*UG** is more dependent on RRF. The reaction mixture was identical to that of Fig 10A except that Mg^2+^ concentration was 7 mM to observe ribosome releasing activity of RRF more clearly. Note that requirement for RRF is clearer under the condition (7 mM Mg^2+^) where non-enzymatic release of ribosomes from mRNA is harder than that in 4 mM Mg^2+^.

**Fig 11:**
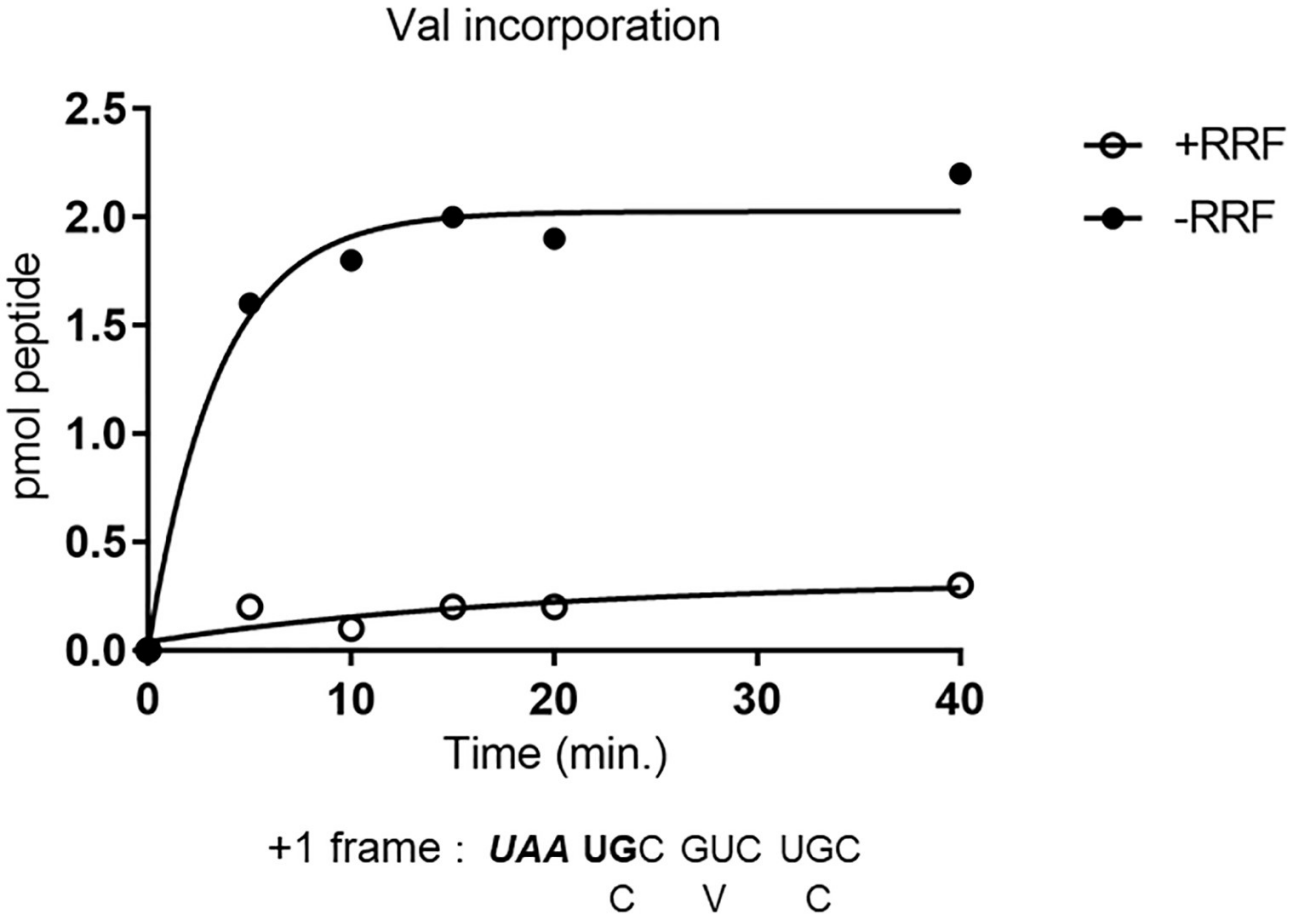
RRF prevents wrong downstream reading in frame with *UAA*; RRF releases ribosomes from *UAA* of *UAA*UG. Downstream reading in frame with ***UAA*** of the junction sequence was measured by [^14^C]-Valine incorporation coded for by triplet GUC which was in frame with ***UAA***. The components of the reaction mixture were identical to that of Fig 9 except for labeled amino acid (valine).

Here, we studied translational coupling through *in vitro* experiments using 027mRNA [45] having the junction sequence ***UAA*UG** without an SD sequence nearby (Fig 8). The u-ORF (shown in italic) contains triplets *UUU* and *UUC* coding for phenylalanine. Therefore, using this mRNA with the PURE system [46], we expect phenylalanine incorporation into polypeptides as indication of upstream reading. For downstream reading, there are two possibilities. First, in the presence of RRF, ribosomes are released from ***UAA*** by RRF and bind to ***A*UG** of ***UAA*UG** on the same mRNA. The ribosome will then read triplets CGU (arginine) followed by CUG (leucine) because they are in frame with ***A*UG** of the junction sequence. Second, in the absence of RRF, ribosomes will remain on ***UAA*** and will start reading from the next codon, **UG**C (cysteine), followed by GUC (valine) as observed by Ryoji *et al* [15]. Therefore, for downstream reading, we expect leucine (0 frame, the ***A*UG** frame) or valine (+1 frame) incorporation in the presence or absence of RRF, respectively. These expectations were met as shown in Figs 9–11.

As shown in Fig 9, phenylalanine incorporation in the presence of RRF (open circles) was much higher than in the absence of RRF (closed circles). The data support the hypothesis that RRF releases the ribosomes at ***UAA*** of ***UAA*UG**, which allow them to be used in the next cycle of u-ORF translation. In the absence of RRF, the reduced incorporation of phenylalanine is due to reduced number of recycled ribosomes.

What about the downstream reading? As shown in Fig 10A, RRF induced additional leucine incorporation (0 frame reading starting from ***A*UG**, see the sequence below the figure) (open circles). This indicates that ribosomes which have finished u-ORF translation at ***UAA*** (+1 frame) initiated downstream reading from ***A*UG** (0 frame). However, even in the absence of RRF (closed circles), comparable amounts of leucine were incorporated. This is because some of the ribosomes at ***UAA*** were released from mRNA non-enzymatically at low (4 mM) Mg^2+^ and rebound to ***A*UG** of ***UAA*UG** resulting in leucine incorporation. Therefore, one would expect that leucine incorporation in the absence of RRF should be reduced at higher Mg^2+^ concentration. This is because Mg^2+^ increases the affinity of RNA to ribosomes [47]. Indeed, as shown in Fig 10B, leucine incorporation in the absence of RRF (closed circles) became much less at 7 mM Mg^2+^ compared with that at 4 mM Mg^2+^ (Fig 10A). Consequently, the dependency of leucine incorporation on RRF became more distinct at high Mg^2+^.

In the case of plus one frame reading of the downstream region (Fig 11), labeled valine was incorporated in the absence of RRF (closed circles). In fact, the sequence of 027mRNA codes for valine only when ribosomes translate after the junction sequence in frame with ***UAA*** (+1 frame). This indicates that ribosomes remaining at the junction sequence ***UAA*UG** did not recognize the ***A*UG** as an initiation codon. In the presence of RRF (open circles), no significant amount of valine was incorporated because ribosomes were released by RRF at ***UAA*** and re-started reading from ***A*UG** of the junction sequence. In these experiments the Mg^2+^ concentration was 4 mM which is unfavorable for the RRF reaction due to non-enzymatic release of ribosomes at lower Mg^2+^. Even in this unfavorable condition, RRF exerted its effect of releasing ribosomes from mRNA resulting in decreased incorporation of valine.

### Complete ribosome splitting is not necessary for the release of ribosomes from mRNA

It has been assumed that the ribosome splitting is a part of ribosome recycling because subunits have been the major player of the initiation which follows the recycling step.

However, in 2015, an *E*. *coli* strain which contains 70S ribosomes that do not split was reported [40]. This strain demonstrates that complete ribosome splitting is not essential for cell growth. In this section, we examined whether the non-dissociable ribosomes (ribo-T) function at the junction sequence, ***UAA*UG**, similarly to the normal ribosomes in the presence and absence of RRF *in vitro*.

**Fig 12:**
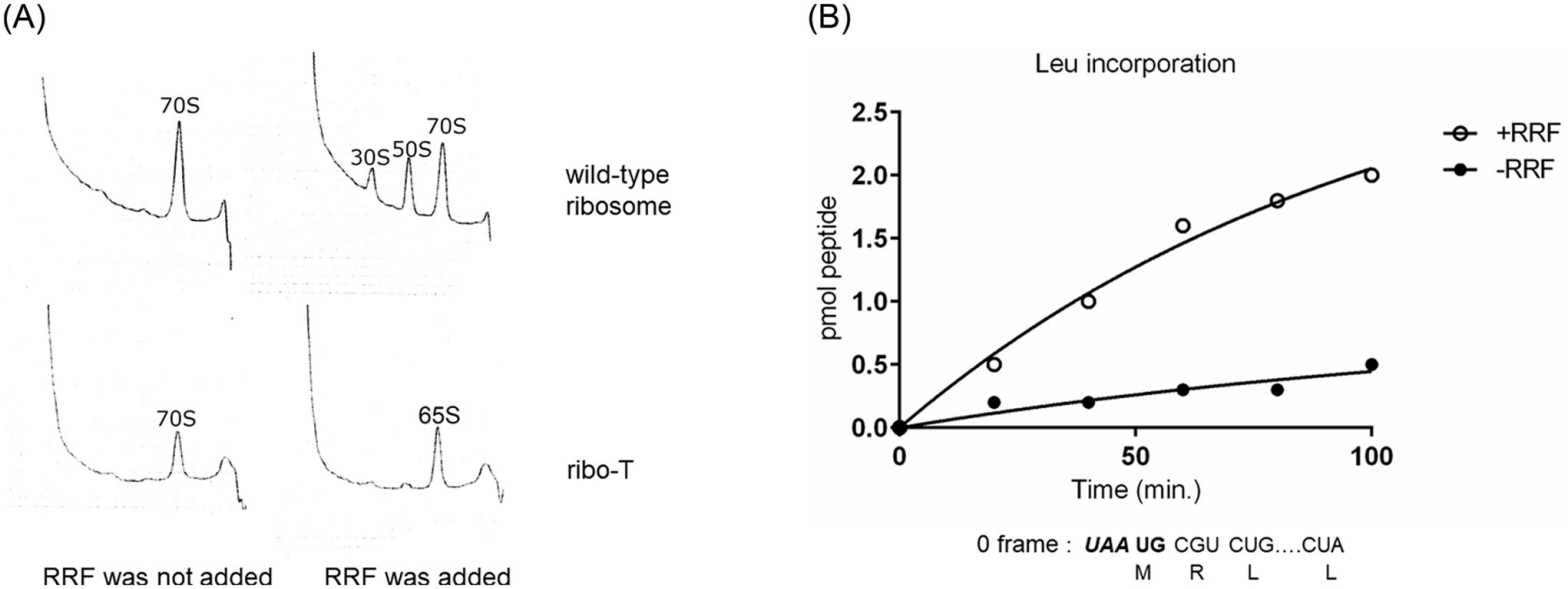
Complete ribosome splitting is not needed for translational coupling. **A** ribo-T is not split while wild-type 70S ribosomes are split into 30S and 50S subunits in the presence of RRF. Upper figures show wild-type ribosome sedimentation and lower figures show that of ribo-T as described in Experimental procedures. Mg^2+^ concentration was 4 mM. **B** Complete splitting of ribosome by RRF is unnecessary for translational coupling; ribo-T functions in a similar manner to wild-type ribosomes. Incorporation of [^14^C]-Leucine coded for by CUG and CUA codons in frame with ***A*UG** was measured as in Fig 10B. The reaction mixture contained 7 mM Mg^2+^ where RRF requirement was more distinctly observed.

It is clear from Fig 12A that, even in the presence of RRF, ribo-T was unable to dissociate into 30S and 50S subunits but converted to 65S particles [40]. On the other hand, the wild-type ribosomes were dissociated into 50S and 30S subunits in the presence of RRF. Using the *in vitro* translational coupling system as described above, we show that ribo-T is released from mRNA by RRF at ***UAA*** of ***UAA*UG** as shown in Fig 12B.

As shown in this figure, in the presence of RRF (open circles), the incorporation of labeled leucine (***A*UG** frame) by ribo-T greatly increased compared with that in the absence of RRF (closed circles). This is similar to the observation in Fig 10B with wild-type ribosomes. This indicates that ribo-T was released from the mRNA at ***UAA*** of ***UAA*UG** by RRF and bound to ***A*UG** without complete splitting. We conclude that complete ribosome splitting is not an obligatory step during the ribosome recycling.

## Discussion

### All *in vivo* and *in vitro* observations are consistent with RRF-dependent release of ribosomes from mRNA during termination process—Prevention of unwanted protein synthesis by RRF

The role of RRF during the normal translation termination process *in vivo* has remained obscure despite extensive biochemical work on RRF. In this paper, we chose the translational coupling system without the SD sequence near the junction sequence as a model system to elucidate this problem. We conclude from these studies that the major function of RRF during the termination process is to release mRNA from the post termination complex (PoTC). We consider that the term “release” includes two stages, release to the immediate vicinity of the mRNA first, followed by the second stage where the ribosomes are far away from the mRNA. In the first stage, the released ribosomes are different from the free ribosomes in that they are close enough to the mRNA to rebind selectively to it without the SD sequence. In the translational coupling discussed in this paper, we deal mostly with the first stage of the release.

Here, we discuss the *in vivo* experiment (Fig 3 and Table 1) most directly indicating the release of ribosomes from mRNA by RRF. In the absence of tsRRF, at the non-permissive temperature (39°C), a much higher proportion of ribosomes from the u-ORF were engaged in downstream reading than those in the presence of RRF at the permissive temperature (28°C) (Table 1, All frames). Since ribosomes must stay on the mRNA for translation of downstream region, RRF-dependent release of ribosomes from mRNA of the PoTC would decrease the total number of ribosomes that read the downstream region. Saito et al., [48] reported that, upon RRF depletion by repressing its transcription, 70S ribosomes accumulated in 3’-UTR with ribosome profiling. However, when a reporter gene was connected downstream, they did not detect re-initiation of translation by these ribosomes. In contrast, we observed unscheduled translation as described in Fig 3 and the previous communication [16]. Although the reason of this discrepancy is not clear, it may be that the residual amount of RRF still remained after RRF depletion because protein synthesis remained as much as 40% [48]. It should be emphasized that, at the non-permissive temperature, over-all protein synthesis in LJ14 is severely reduced due to the lack of ribosome recycling for a new round of protein synthesis [49].

The *in vitro* experiments using wild-type RRF and the mRNA designed to study translational coupling (Fig 8) also suggested the ribosome releasing function of RRF. In this system, in the presence of RRF, ribosomes began to read from ***A*UG** of the junction sequence ***UAA*UG**. This means that the ribosome must be released from ***UAA***. The released ribosomes must be rebound to mRNA to read the rest of the mRNA in frame with ***A*UG** (leucine incorporation, Fig 10A and 10B). In contrast, in the absence of RRF, the ribosome remained on ***UAA*** and translated downstream in frame with ***UAA*** (valine incorporation, Fig 11).

The release of ribosomes from PoTC results in the prevention of the synthesis of unwanted protein such as the peptide containing valine as shown in Fig 11. This is because this peptide is not scheduled to be synthesized and would have a lethal effect. Prevention of unscheduled translation is a consequence of the function of RRF; the release of mRNA from PoTC.

**Fig 13:**
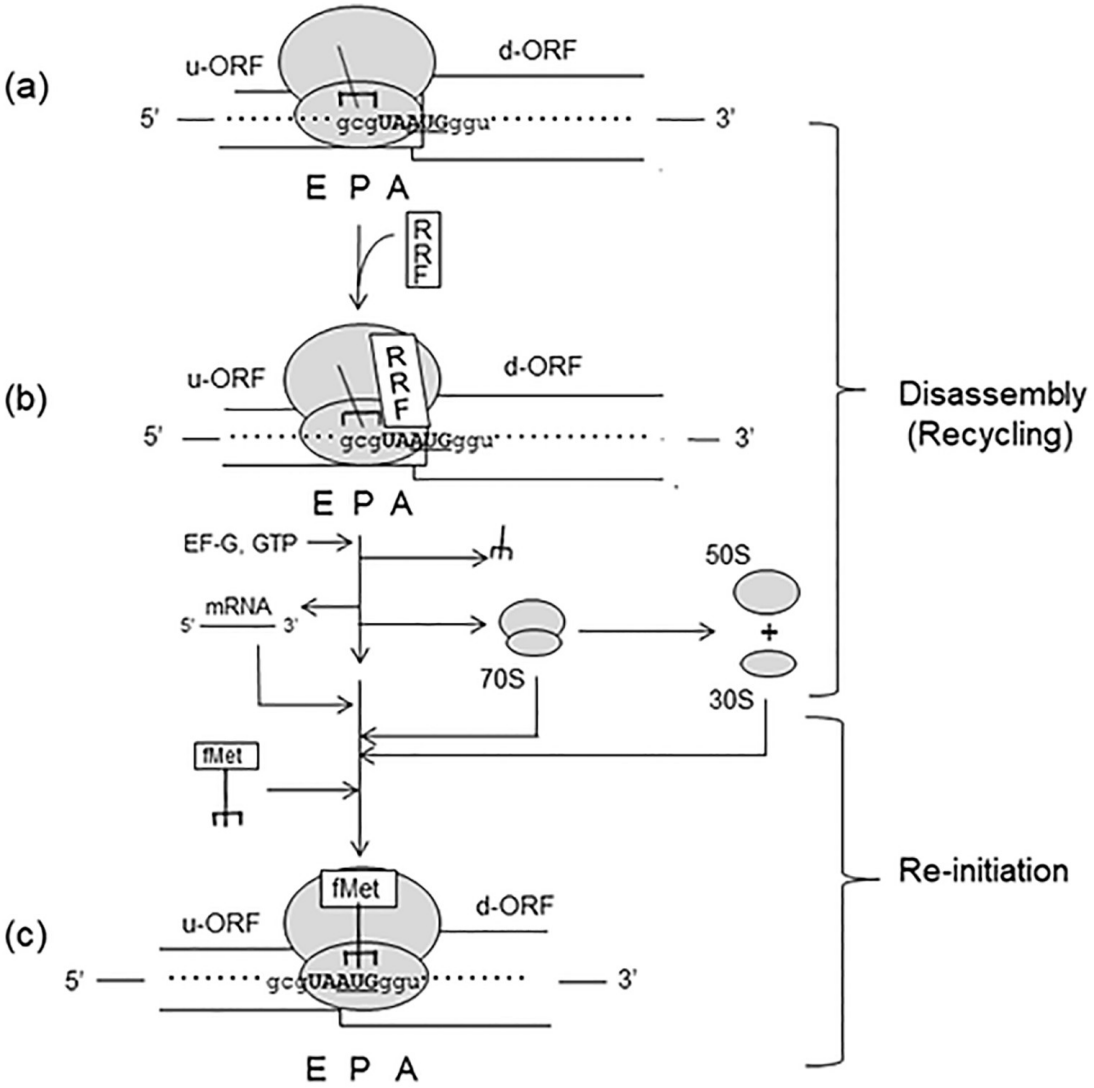
RRF’s function in translational coupling without SD. Complex (a); Post Termination Complex (PoTC) at the junction sequence of translationally coupled ORFs. The junction sequence **UAAUG** is shown in bold. Complex (b); RRF is bound on the ribosome at A/P-site. Complex (c); Completed 70S initiation complex. Process from (b) to (c); The ribosome and deacylated tRNA are released from the mRNA by RRF and EF-G action. The ribosome may or may not split into subunits. Both the 70S ribosomes and the ribosome subunits that engaged in the reading of the u-ORF may participate in downstream reading. The released 70S ribosomes or the dissociated ribosome subunits bind to the adjacent **AUG** on the same mRNA with the help of fMet-tRNA.

When ribosomes translating the u-ORF reach the junction sequence, the tRNA carrying the nascent u-ORF gene product is bound to the last codon of u-ORF at the P-site. The u-ORF peptide is then released by the release factor attracted by the termination codon at the A-site. The resulting ribosomal complex has the tRNA bound mostly to P/E site (some at the P/P site [24]). In Fig 13, we summarize RRF’s function *in vivo* during natural termination process represented by our model translational coupling system. In complex (a) of this figure, the A-site of PoTC is empty because the release factor which had hydrolyzed the ester bond between tRNA and the u-ORF protein was released with the help of RF3 [50,51]. This will give RRF the chance to bind at the A/P site [22,23], resulting in complex (b) formation. In the step from complex (b) to (c), tRNA is released from the P-site [24], the ribosome is released from the mRNA without splitting due to the action of RRF together with EF-G and GTP [1,41]. The released 70S ribosomes may or may not dissociate into 30S and 50S subunits by RRF and EF-G (or IF3 alone). At this point the recycling step is completed. The exact molecular mechanism of release of RRF and EF-G from PoTC is to be elucidated. In complex (c), because of the proximity of **AUG** to **UAA**, some of the released ribosomes which are in the immediate vicinity of mRNA are re-bound to **AUG** of **UAAUG** with formyl methionyl tRNA. Re-initiation of translation starts and the reporter *lacZ* gene is expressed. It should be noted that there are junction sequences with an SD sequence of the d-ORF [52]. However, such a system is not suitable for studying the behavior of ribosomes at the end of the natural ORF without an SD sequence around the termination codon.

### Is the splitting of 70S ribosome essential for disassembly of PoTC?

Although our *in vivo* data presented in this paper do not directly exclude the possibility of 30S/mRNA complex as an intermediate of disassembly of PoTC, we avoid such complex. This is because none of the ribosomes from PoTC without SD near the termination triplet had 16S ribosomal RNA only. Additionally, none of the PoTC had visible poly-30S ribosomal subunits (Figs 5 and 9 of [24]) [28]. The 30S/mRNA complex have been observed only with those with nearby SD (for example [29]).

Furthermore, complete splitting of ribosomes is not necessary for translational coupling because ribosomes with tethered subunits [40] still function like wild-type ribosomes at the junction sequence. The observation *in vitro* (Fig 12B) showed that ribosome release from ***UAA*** of the junction sequence ***UAA*UG** and rebinding to ***A*UG** still occurred (resulting in leucine incorporation in frame with ***A*UG**) without complete separation of subunits. This is consistent with the notion that initiation with 70S ribosomes [53], rather than subunits, is commonly involved in prokaryotic protein synthesis [54]. Translational coupling, a common ribosomal activity [37], must also be occurring in this mutant [40]. These observations support scheme not involving 30S/mRNA intermediate. In conclusion, we presented data to emphasize that the major *in vivo* function of RRF in the normal termination process is to release ribosomes from the PoTC without splitting.

### Effects of high culture temperature in the absence of RRF

In this paper, we used a tsRRF mutant to eliminate the function of RRF *in vivo*. With this mutant, high culture temperature and simultaneous loss of RRF created an unusual situation. Under these unique conditions, ribosomes at junction sequences exhibited unexpected behavior. The frameshift of ribosomes on mRNA to all frames (temperature-dependent frameshift) occurred (Figs 3 and 4). We should add that this frameshift is limited to the mRNA-bound ribosomes without peptidyl groups. Such ribosomal complexes are imaginable when RRF is inactivated because the substrate of RRF is the ribosomes with one tRNA without a peptidyl group mostly in the P/E site [24]. Because of the absence of the peptidyl group which stabilizes ribosomes on the mRNA, the ribosome would slide on the mRNA resulting in the thermal frameshift.

**Fig 14:**
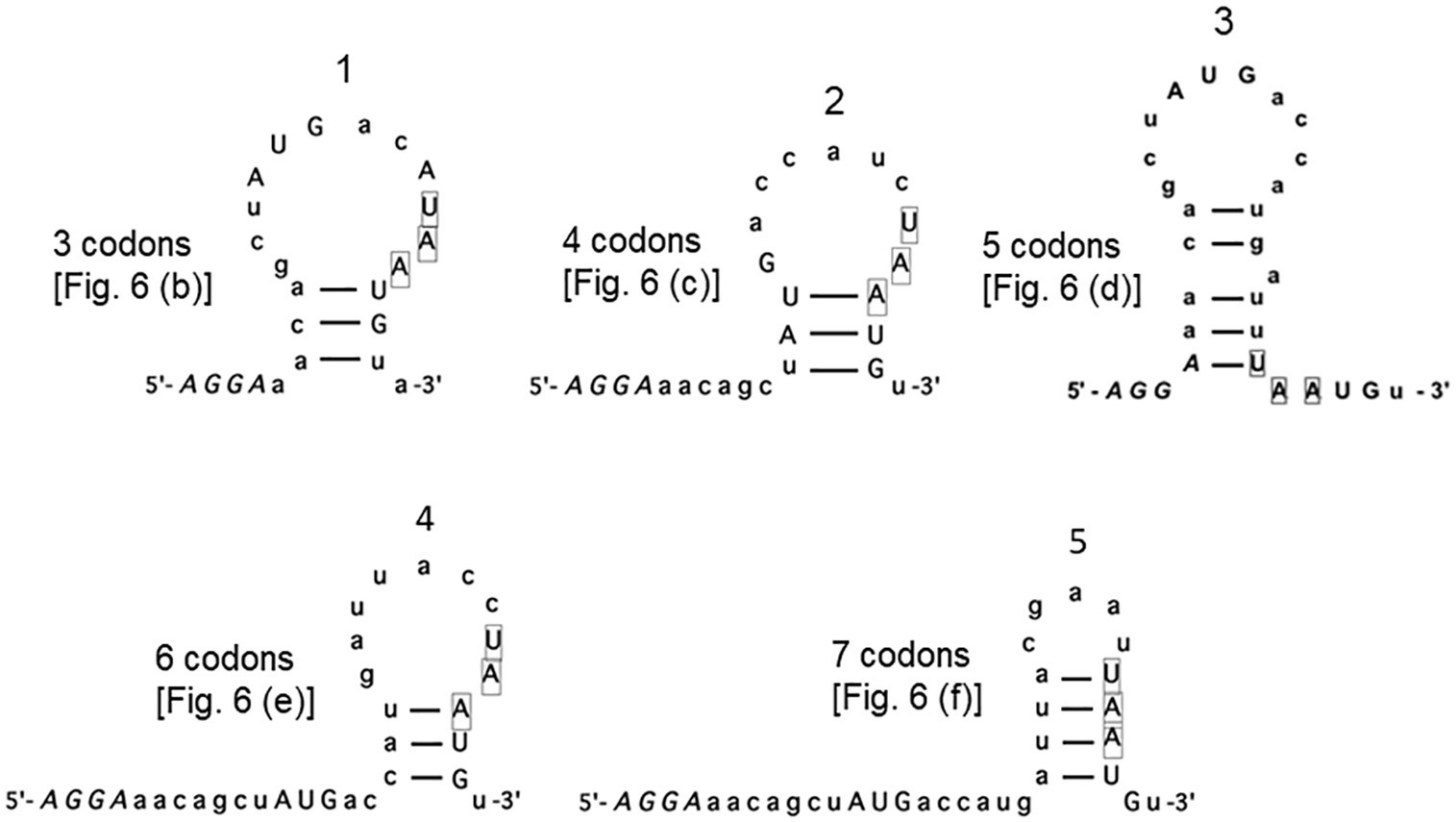
Possible mRNA secondary structures around the junction sequence at the non-permissive temperature of tsRRF. Stem and loop structures were constructed according to Freier *et al*. [56]. The SD sequence (5’-*AGGA*-3’), initiation and termination codons are represented by capital letters. Termination codons of u-ORF are boxed for clarity. Bars between nucleotides represent Watson-Crick type base-pairing.

The high expression of d-ORF in Fig 6 panel (d) in the absence of RRF due to high culture temperature suggests that ribosome-bound mRNA can take on a secondary structure at the junction region. With short u-ORF, such secondary structures would be able to shorten the distance between SD and the termination codon of u-ORF. In the case of Fig 6 panel (d), by base-pairing between front and rear parts of the u-ORF, the SD sequence (*AGGA*) and the termination codon UAA would adjoin each other (hairpin structure 3 of Fig 14). This short distance between SD and UAA would stimulate u-ribosomes on UAA to bind to AUG of UAAUG resulting in high d-ORF translation. However, the idea of secondary structure formation is against the general belief that mRNA bound to the ribosome at the channel of the 30S subunit [55] cannot take on a secondary structure. Perhaps our assumption (the secondary structure formation of ribosome-bound mRNA) is only possible when the ribosome does not have a peptidyl group as in the case of PoTC.

### The influence of nearby SD on the role of RRF as a ribosome-releasing factor

During our studies, we noticed that the SD sequence of short u-ORF has a strong influence on the behavior of ribosomes in translational coupling. The SD of the short u-ORF attracted ribosomes which were released by RRF from the termination codon. This caused the ribosomes to bind back to the u-ORF initiation codon resulting in loss of d-ORF reading (for example, Fig 6 panels (b), (c) and (d)). Furthermore, nearby SD pulled even the mRNA-bound ribosomes that resulted from inactivation of temperature sensitive RRF (Fig 6 panel (b)). This is consistent with the report that SD has an inhibitory effect on movement of mRNA-bound translating ribosomes [57]. Several reports indicate that SD sequences facilitate frameshift at the region involving slippery sequences [58–60]. This is most likely due to the pulling force of SD sequences on mRNA-bound translating ribosomes.

We showed the lack of downstream reading when SD of u-ORF was near the junction sequence as discussed above. This indicates that the use of short mRNA with SD (quoted in the recent review [61]) should be avoided for studies of RRF action at the end of natural ORF. The only “natural” short mRNA (mini-gene) with SD we could find is mRNA encoding a dipeptide related to the lambda phage [62]. The expression of mini-genes encoding less than seven amino acids is toxic to growth of the cell due to accumulation of peptidyl-tRNA and depletion of the tRNA [63]. The toxicity increases as the length of mini-gene shortens [63]. Zavialov et al., [8] reported that the original interaction between SD and anti-SD remained intact in the PoTCs *in vitro* when the lengths of ORFs were shorter than 6 codons, making a small loop containing the SD sequence. Their results are consistent with our idea that SD pulls back ribosomes finishing translation of short u-ORF without d-ORF translation. These *in vitro* and *in vivo* studies predict that the use of short ORF would make it impossible to observe the release of ribosomes from the mRNA. In support of this finding, a cryo-electron microscopic study with short mRNA (4 codons ORF) containing SD showed that the mRNA is never released from the PoTC [29]. The *in vitro* studies with various short mRNAs suggest that the SD sequence of the short ORF pulls back ribosomes from the termination codon of the ORF toward the SD and binds them to the initiation codon of the ORF. Therefore, the behavior of the ribosome after finishing reading such a short ORF with SD may not necessarily be the same as that of ribosomes released from the termination codon of natural ORFs without nearby SD. Here, we developed a new *in vitro* translational coupling system with 027mRNA which was long enough to abolish the effect of SD on ribosomal behavior at the termination step. Using this system, we showed that RRF released ribosomes at the termination codon of the mRNA *in vitro*. The studies presented in this paper show the important function of RRF, release of ribosomes from mRNA, which could not be observed in the *in vitro* studies with short ORFs containing SD.

There are translational couplings with [36] and without SD of d-ORF [39,42]. Our results stated above are partially consistent with the recent publication by Saito et al., [48] which shows that loss of RRF does not affect translational coupling. This is because they used gene pairs having SD motifs (for example, AGGA) in the junction regions. We think that loss of RRF activity of releasing ribosomes from termination codon is compensated by pulling force of SD to ribosomes remaining at the termination codon. In the current study, we showed that, with the help of the SD of short u-ORF near the junction sequence, u-ribosomes could initiate translation of d-ORF even in the absence of RRF (Fig 7). On the contrary, in the absence of nearby SD, RRF is essential to initiate downstream translation from only the AUG of d-ORF (Fig 3) [39].

## Materials and methods

### Bacteria

*Escherichia coli* JM109 (*recA1*, *endA1*, *gyrA96*, *thi*, *hsdR17*, *mcrA*^*-*^, *supE44*, *relA1*, *Δ(lac-proAB)/F’traD36*, *proAB*^*+*^, *lacI*^*q*^, *lacZΔM15*) was obtained from Takara Bio Inc. and used as recipient of the plasmids for transformation. The *E*. *coli* strain LJ14/*F’lacI*^*q*^ [39] is designated simply as LJ14 in this text.

### Materials

Restriction endonucleases, DNA ligase, TaKaRa Ex Taq DNA polymerase and PrimeSTAR^®^ Mutagenesis Basal Kit were purchased from Takara Bio Inc. The plasmids pUC18, pKK223-3 and pMC1871 DNAs were purchased from Pharmacia Biotech.

### Plasmids

Plasmid pUC18 is a vector carrying the *lac* promoter and α-peptide sequence of the *lac*Z gene into which a 54 base pair multiple cloning site (MCS) polylinker sequence is inserted. Plasmid pKK223-3 is a vector carrying the *tac* promoter and a 33-base pair MCS polylinker sequence. Plasmid pMC1871 has the *lac*Z gene lacking its promoter, the ribosome-binding site, and the twenty-seven 5’-terminal nucleotides including the initiation codon AUG of the *lac*Z gene, which is represented as *lac*Z(Δ27) in this paper because of the lack of 27 nucleotides. The DNA sequences from the junction sequence through the reporter gene of the plasmids were confirmed using CEQ8000 DNA sequencer (Beckman Coulter, Inc.) or 3130 Genetic Analyzer (Applied Biosystems). Preparations of plasmids used in this study are described in Supplemental Information and their nucleotide sequences are shown in S1 Table.

### Assay of β-galactosidase activity

*E*. *coli* LJ14 cells harboring the plasmid carrying the *lacZ*(Δ27) gene or the *lacZ*(Δ78) gene were grown at 28°C in LB broth (1.0% tryptone (Difco), 0.5% yeast extract, 1.0% sodium chloride) containing 50 μg/ml ampicillin to a cell density (OD_660_) of ~0.13. At that time, the culture was divided into two parts and 2 mM (final concentration) IPTG was added to each part to induce transcription of the *lac* gene. Then, one part was transferred and incubated at 39°C for an additional 2 hours. The other part was kept at 28°C. At the indicated times, an aliquot (100 μl) was taken and the β-galactosidase activity was measured according to Miller [64] except that an OD_660_ of cell density was used instead of OD_600_. Miller units were calculated using the conversion factor, OD_660_/OD_600_ = 0.57. The β-galactosidase activity was expressed as means of two or three experiments with ± SE shown by error bars. When the size of the bar was smaller than that of the circles the bar was not shown in the figure.

### *In vitro* translation system using wild-type and tethered ribosomes

The reaction mixtures (210 μl) contained 4 or 7 mM magnesium acetate, 150 mM potassium chloride, 20 mM Tris-HCl pH 7.6, 300 μM spermidine, 15 mM putrescine, 1 mM dithiothreitol, 1 mM GTP, 2 mM ATP, 10 mM phosphoenolpyruvate (PEP), 0.07 mg/ml PEP Kinase, 5 μM EF-G, 1 μM of each initiation factor (IF1, IF2 and IF3), 10 μM EF-Tu, 5 μM EF-Ts, 140 U of aa-tRNA synthetase, 1 μM formyl methionine transferase, 850 μM 10-formyltetrahydrofolate, 2 μM RF1, 2 μM RF3, 20 μM of each amino acid required for the translation of 027mRNA [45], 10 μM tRNAs mixture, 0.5 μM ribosome, 2.5 μM 027mRNA and 5 μM RRF, when present. The reaction mixtures were incubated at 37°C and after 0, 5, 10, 15, 20 and 40 minutes of incubation, 30 μl of the reaction mixture was spotted on a 1 cm x 1 cm 3MM filter and dropped in 5% TCA and 0.25% sodium tungstate for at least 1 hour at room temperature. The TCA/sodium tungstate mixture was changed and boiled for 10 minutes, washed three times with the same mixture, one time with ethanol/ether (v/v) and one time with ether. The filters were dried and the radioactivity was measured with a scintillation counter. For measuring phenylalanine incorporation, unlabeled phenylalanine was replaced in the above reaction mixture with 0.2 μM [^3^H]-phenylalanine and 19.8 μM of unlabeled phenylalanine (final concentration was 20 μM). 20 pmols of phenylalanine gave 2,650 CPM. This value was used to calculate the pmol of phenylalanine incorporated. For measuring leucine incorporation, [^14^C]-leucine was added in the reaction mixture to a final concentration of 20 μM. Twenty pmols of leucine corresponded to 13,447 CPM. For measuring valine incorporation, [^14^C]-valine was added in the reaction mixture to a final concentration of 25 nM. Twenty pmols of valine corresponded to 15,600 CPM.

### Sedimentation analysis

Wild-type ribosomes and ribo-T were each incubated at 30°C for two minutes with 5 μM EF-G and 5 μM IF3 in the presence or absence of 5 μM RRF. After incubation, the reaction mixture was placed on ice to stop the splitting reaction and loaded onto a 4.5 ml sucrose gradient (15–35%) and spun for 3 hours and 45 minutes at 40,000 RPM using a Beckman SW50.1 rotor.

## Supporting information

S1 Table Plasmids used in this study. (PDF)
