# Supplementary material for: Role of ribosome recycling factor in natural termination and translational coupling as a ribosome releasing factor
Source: PLoS One. 2023 Feb 24;18(2):e0282091. doi: 10.1371/journal.pone.0282091 (PMC9955659; doi:10.1371/journal.pone.0282091)
Supplement: S1 Table — (PDF) [file pone.0282091.s001.pdf]

Supporting information

S1 Table. Plasmids used in this study

Plasmid	Nucleotide sequence	Fig and panel
pIY10	5'- aggaaacagctATG$(N)_{88}$acgcgTAATGtacgta・*lacZ*(Δ27) -3'	Fig 1 (A), Fig 3 (b)
pIY11	5'- aggaaacagctATG$(N)_{88}$acgcgTAATGctacgta・*lacZ*(Δ27) -3'	Fig 3 (c)
pIY12	5'- aggaaacagctATG$(N)_{88}$acgcgTAATGcctacgta・*lacZ*(Δ27) -3'	Fig 3 (d)
pIY20	5'- aggaaacagctATG$(N)_{88}$acgcgTAATAtacgta・*lacZ*(Δ27) -3'	Fig 3 (e)
pIY21	5'- aggaaacagctATG$(N)_{88}$acgcgTAATActacgta・*lacZ*(Δ27) -3'	Fig 3 (f)
pIY22	5'- aggaaacagctATG$(N)_{88}$acgcgTAAtacctacgta・*lacZ*(Δ27) -3'	Fig 3 (g)
pIY27U	5'- aggaaacagctATG$(N)_{27}$cccggggatccc・*lacZ*(Δ27) -3'	Fig 3 (a)
pIY78K	5'- aggaaacagctATGtacgta・*lacZ*(Δ78) -3'	Fig 6 (a), Fig 7 (a)
pIY78K2	5'- aggaaacagctATGacATGccgtacgta・*lacZ*(Δ78) -3'	Fig 7 (d)
pIY100	5'- aggaaacagctATG$(N)_{88}$acgcgTAATGggtc$(N)_{30}$aatacgta・*lacZ*(Δ78) -3'	Fig 4 (a) - (e)
pIY101	5'- aggaaacagctATG$(N)_{88}$acgcgTAATGggtc$(N)_{30}$tacgta・*lacZ*(Δ78) -3'	Fig 4 (f) - (j)
pIY102	5'- aggaaacagctATG$(N)_{88}$acgcgTAATGggtc$(N)_{30}$atacgta・*lacZ*(Δ78) -3'	Fig 4 (k) - (o)
pIY200	5'- aggaaacagctATG$(N)_{88}$acgcgtcATGccccTAAcctacgta・*lacZ*(Δ27) -3'	Fig 5 (a)
pIY202	5'- aggaaacagctATG$(N)_{88}$acgcgtATGccTAActacgta・*lacZ*(Δ27) -3'	Fig 5 (b)
pIY203	5'- aggaaacagctATG$(N)_{88}$acgcgtcATGcTAAcctacgta・*lacZ*(Δ27) -3'	Fig 5 (c)
pIY206	5'- aggaaacagctATG$(N)_{88}$acgcgtcATGAcctacgta・*lacZ*(Δ27) -3'	Fig 1 (B), Fig 2 (c)
pIY209	5'- aggaaacagctATG$(N)_{88}$acgcgTAATGtacgta・*lacZ*(Δ27) -3'	Fig 2 (a), Fig 5 (d)
pIY210	5'- aggaaacagctATG$(N)_{88}$acgcgTAAATGtacgta・*lacZ*(Δ27) -3'	Fig 5 (e)
pIY211	5'- aggaaacagctATG$(N)_{88}$acgcgTAAcATGtacgta・*lacZ*(Δ27) -3'	Fig 5 (f)
pIY214	5'- aggaaacagctATG$(N)_{88}$acgcgTAAccccATGtacgta・*lacZ*(Δ27) -3'	Fig 5 (g)

pIY215	5'- aggaaacagctATG(N)$_{88}$acgcgtcATAActacgta・*lacZ*(Δ27) -3'	Fig 2 (e)
pIY0206	5'- aggaaacagctATT(N)$_{88}$acgcgtcATGAcctacgta・*lacZ*(Δ27) -3'	Fig 2 (d)
pIY0209	5'- aggaaacagctATT(N)$_{88}$acgcgTAATGtacgta・*lacZ*(Δ27) -3'	Fig 2 (b)
pIY1110	5'- aggaaacagctATGacATAATGtacgta・*lacZ*(Δ78) -3'	Fig 6 (b), Fig 7 (b)
pIY1130	5'- aggaaacagctATGacATGAaatacgta・*lacZ*(Δ78) -3'	Fig 7 (c)
pIY2180	5'- aggaaacagctATGaccatcTAATGtacgta・*lacZ*(Δ78) -3'	Fig 6 (c)
pIY2200	5'- aggaaacagctATGaccatgattTAATGtacgta・*lacZ*(Δ78) -3'	Fig 6 (d)
pIY2300	5'- aggaaacagctATGaccatgattaccTAATGtacgta・*lacZ*(Δ78) -3'	Fig 6 (e)
pIY2400	5'- aggaaacagctATGaccatgattacgaatTAATGtacgta・*lacZ*(Δ78) -3'	Fig 6 (f)

Capital letters in the nucleotide sequences represent the initiation and termination codons. Double underlines represent the Shine-Dalgarno sequence (5'-agga-3'). Underlines represent the recognition sequences for the restriction enzyme *Mlu*I (5'-acgcgt-3'), *Sna*BI (5'-tacgta-3') and *Sma*I (5'-cccggg-3'). N represents a nucleotide and numbers next to the bracket represent the number of nucleotides in the bracket. $(N)_{88}$ represents the region containing the MCS (multi cloning site) of pUC18 and the distal part of coat gene of GA phage [1]. $(N)_{30}$ is the sequence 5'-tcaaagcaaaacacaaggaaaacctcgcga-3': the proximal part of the lysis gene downstream of the coat-lysis junction sequence of GA phage [2]. $(N)_{27}$ is the sequence 5'-accatgattacgaattcgagctcggta-3': the proximal part of $\alpha\alpha$-peptide gene of pUC18 [3]. The *lacZ*(Δ78) gene lacks the 78 proximal nucleotides of the *lacZ* gene. Deletion of the 26 N-terminal amino acid residues of β-galactosidase does not affect its enzyme activity [4].

References

1. Inokuchi Y, Hirashima A, Sekine Y, Janosi L, Kaji A. 2000. Role of ribosome recycling factor (RRF) in translational coupling. EMBO J 19:3788-3798.
2. Inokuchi Y, Takahashi R, Hirose T, Inayama S, Jacobson AB, Hirashima A. 1986. The Complete Nucleotide Sequence of the Group II RNA Coliphage GA. J Biochem 99:1169-1180.
3. Norrander J, Kempe T, Messing J. 1983. Construction of improved M13 vectors using oligodeoxynucleotide-directed mutagenesis. Gene 26:101-106.
4. Fowler AV, Zabin I. 1983. Purification, Structure, and Properties of Hybrid β-Galactosidase Proteins. J Biol Chem 258:14354-14358.
